# Evolution of sex determination and heterogamety changes in section *Otites* of the genus *Silene*

**DOI:** 10.1038/s41598-018-37412-x

**Published:** 2019-01-31

**Authors:** Veronika Balounova, Roman Gogela, Radim Cegan, Patrik Cangren, Jitka Zluvova, Jan Safar, Viera Kovacova, Roberta Bergero, Roman Hobza, Boris Vyskot, Bengt Oxelman, Deborah Charlesworth, Bohuslav Janousek

**Affiliations:** 1 Department of Plant Developmental Genetics, Institute of Biophysics of the Czech Academy of Sciences, Kralovopolska 135, 61265 Brno, Czech Republic; 2 Department of Biological and Environmental Sciences, University of Gothenburg, 40530 Gothenburg, Sweden Sweden; 3 Centre of the Region Haná for Biotechnological and Agricultural Research, Institute of Experimental Botany of the Czech Academy of Sciences, 78371 Olomouc, Czech Republic; 4 Institute for Biological Physics, University of Cologne, Zülpicher Straße 77, Cologne, Germany; 5 Institute of Evolutionary Biology, EH9 3FL University of Edinburgh, Edinburgh, UK

## Abstract

Switches in heterogamety are known to occur in both animals and plants. Although plant sex determination systems probably often evolved more recently than those in several well-studied animals, including mammals, and have had less time for switches to occur, we previously detected a switch in heterogamety in the plant genus *Silene*: section *Otites* has both female and male heterogamety, whereas *S*. *latifolia* and its close relatives, in a different section of the genus, *Melandrium* (subgenus *Behenantha*), all have male heterogamety. Here we analyse the evolution of sex chromosomes in section *Otites*, which is estimated to have evolved only about 0.55 MYA. Our study confirms female heterogamety in *S*. *otites* and newly reveals female heterogamety in *S*. *borysthenica*. Sequence analyses and genetic mapping show that the sex-linked regions of these two species are the same, but the region in *S*. *colpophylla*, a close relative with male heterogamety, is different. The sex chromosome pairs of *S*. *colpophylla* and *S*. *otites* each correspond to an autosome of the other species, and both differ from the XY pair in *S*. *latifolia*. *Silene* section *Otites* species are suitable for detailed studies of the events involved in such changes, and our phylogenetic analysis suggests a possible change from female to male heterogamety within this section. Our analyses suggest a possibility that has so far not been considered, change in heterogamety through hybridization, in which a male-determining chromosome from one species is introgressed into another one, and over-rides its previous sex-determining system.

## Introduction

Genetic sex-determining systems in plants with separate sexes (dioecious plants) are thought mostly to have evolved via gynodioecy, involving a male sterility mutation in a hermaphroditic or monoecious ancestor as a first step^1^, or perhaps sometimes gradually from a monoecious ancestor, in the “paradioecy pathway”^2–4^. The gynodioecy route is predicted to generate male heterogamety more often than female heterogamety, because male sterility mutations are mostly recessive, consistent with females being the homozygous sex^1^. It has therefore been suggested that species with female heterogamety evolved this state secondarily, from ancestral systems with male heterogamety. Although changes in heterogamety are known in several animal and plant taxa, empirical evidence on the directions of the changes is scanty. However, some species with female heterogamety appear to have evolved without a previous male heterogametic system. For example, in the genus *Fragari*a (*Rosaceae*), subdioecious and dioecious species have female heterogamety, with dominant male sterility alleles in *F*. *virginiana*^5,6^ and *F*. *chiloensis*^7^. The analysis of the genetic determination of male sterility in a gynodioecious species, *F*. *vesca subsp*. *bracteata*, revealed the presence of a dominant nuclear male sterility gene (it is epistatically dominant to a dominant fertility restorer that is also present)^8^. In two other plant genera where multiple species have been studied, *Cotula*^9^ (*Asteraceae*), and *Pistacia*^10,11^ (*Anacardiaceae*), only female heterogamety has been found, again suggesting that there might have been no previous history of male heterogamety. In two further genera with female heterogamety, only single species have been tested (*Distichlis*^12^ in the *Poaceae*, and *Potentilla*^13^ in the *Rosaceae*).

Several animal genera with both male and female heterogamety are known^14^, but there is firm evidence in only two plant genera, *Populus*^15^, in the family *Salicaceae*, and *Silene*^16,17^, in the family *Caryophyllaceae*. In fish, many examples of differences in the chromosome carrying the sex-determining locus in related species have been documented, including in medaka and related species^18,19^ and in cichlids^20–22^, sometimes changing the heterogamety. Similar events have also occurred in *Silene*. Turnovers of sex-determining regions, with no change in heterogamety, have been described in *Fragaria* species, involving inferred repeated translocation of small regions between homeologous chromosomes in octoploid species^23^. In the *Salicaceae*, almost all species are dioecious, and the available data on fossils suggest that dioecy could be as old as 45 My^24,25^, so multiple changes could have occurred during this evolutionary history. The current sex chromosomes in *Populus trichocarpa* are estimated to have evolved only ~6–7 MYA ago, based on divergence between X- and Y-linked sequences^26^, supporting the view that multiple switches in sex determination occurred or even a possibility of independent evolution of dioecy in this lineage of *Salicaceae*, rather than dioecy being ancestral to the family^27–30^. Despite the different localization of sex-determining regions in *Salix* and *Populus* (chromosome 15 and 19), recent work in *S*. *purpurea* suggests a possible common origin of some sex-linked loci in *Salix* and *Populu*s^31^. The sex-linked region in *S*. *purpurea* is estimated to have evolved recently^31^, so it is not possible to test whether female heterogamety was ancestral to *Salicaceae*.

**Figure 1:**
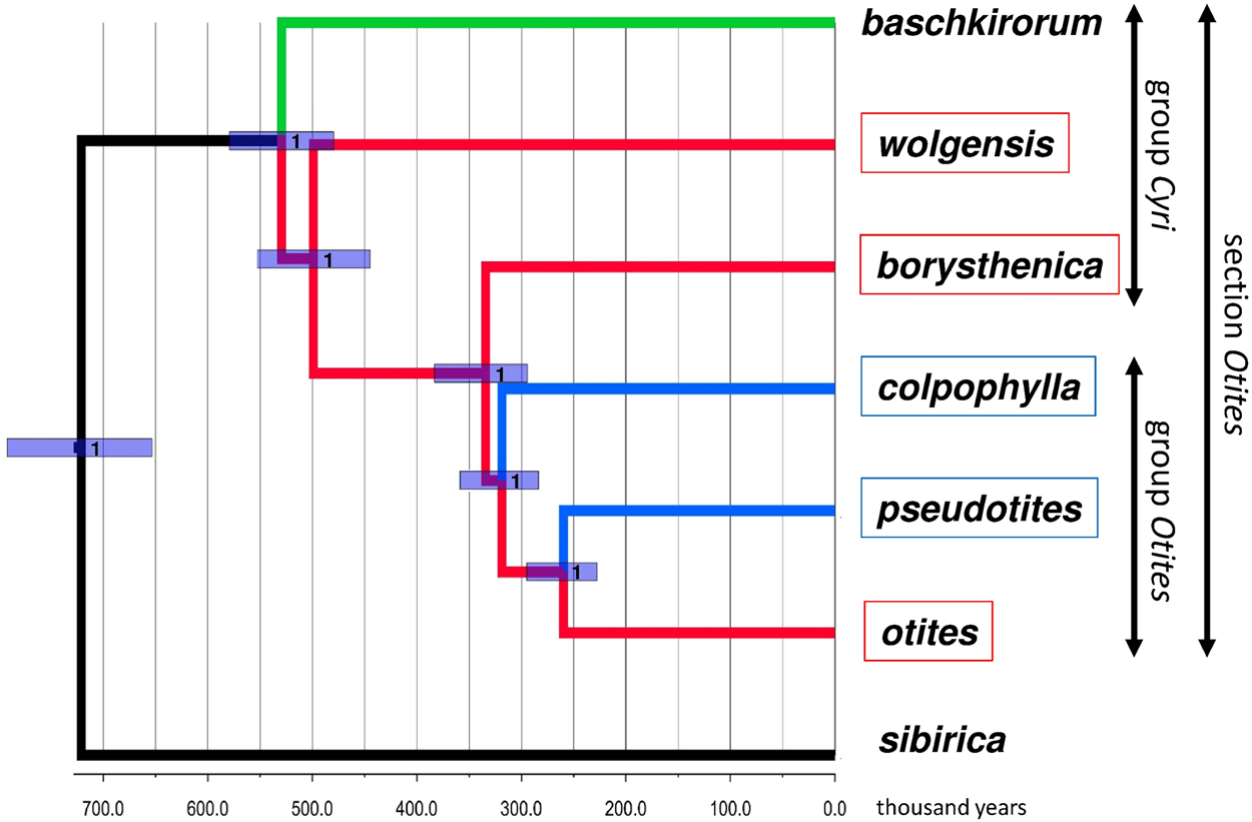
Results of StarBeast phylogenetic analysis of 662 single-copy orthogroup sequences. Species with female heterogamety are indicated by red boxes, and male heterogamety by blue boxes. Female heterogamety in *Silene borysthenica*, and *S*. *otites*, and male heterogamety in *S*. *colpophylla*, were confirmed or inferred from the molecular data presented here, and the male heterogamety in *S*. *pseudotites* and female heterogamety in *S*. *wolgensis* from results of interspecific crosses. The values indicated at the nodes are posterior probabilities. Branch colours indicate the most probable scenario for the evolution of the sex-determining system in section *Otites*. Branches and nodes where female heterogamety is inferred are coloured red, while the branches with male heterogamety are in blue. The branch to *S*. *baschkirorum* is coloured green as its heterogametic state is not known. *Silene sibirica* is not dioecious.

The genus *Silene* (family *Caryophyllaceae*) has a diversity of sexual systems^32^, including naturally occurring cytoplasmic male sterility (gynodioecy), which have been studied in detail in *S*. *vulgaris* and *S*. *nutans*^33–35^. The evolution of dioecy in the genus *Silene* is therefore thought to have involved the gynodioecy-based two-locus pathway. The chromosome number in almost all species of the genus *Silene* is 2n = 24^36^ (although polyploids have been described in several species of the genus *Silene* and are most common among North American species^37^). In contrast to the genus *Fragaria*, all dioecious species of the genus *Silene* so far studied are diploid in natural populations^38,39^. Dioecy in the *Caryophyllaceae* probably evolved within the past 10 million years^17,40–43^. Multiple changes in sex determination are therefore less likely than in the *Salicaceae*, and changes in state can reliably be inferred. In the genus *Silene*, at least three independent origins of genetic sex determination (dioecy and/or subdioecy) have been reported^17^, as well as a switch from XY to ZW, or vice versa, in section *Otites*^17^. In the present study, we identified sex-linked genes in three section *Otites* species (Fig. 1), *S*. *borysthenica* (group *Cyri*), and *S*. *colpophylla* and *S*. *otites* (group *Otites*), allowing us to test (i) whether male heterogamety was ancestral in section *Otites* (which also includes *S*. *pseudotites*), and (ii) whether the chromosomes carrying the sex-determining loci of different dioecious species with the same heterogamety evolved from the same or different autosome pairs, or are likely to represent turnovers in sex determination, and to detect the lineages that underwent switches in heterogamety, potentially helping to test between different hypotheses for the causes of these changes.

The causes of turnovers in sex determination and switches in heterogamety have been classified into three main categories (reviewed by Beukeboom & Perrin^14^). (1) Selectively neutral changes. Recent modelling of finite populations^44,45^ has shown that genetic drift favors the spread and fixation of dominant masculinising or feminising alleles, which can lead to changed sex determination in a population in which a new genotype involved in sex determination has no fitness advantage (for example a dominant female-determining mutation on an X chromosome in an XY system); dominant alleles are therefore predicted to replace ones with lesser dominance. (2) Changes caused by fitness differences between sex phenotypes can be due to a new sex-determining mutation arising closely linked to a gene with a polymorphism for sexually antagonistic alleles (reviewed by van Doorn^46^), or be advantageous due to genetic degeneration of an ancestral non-recombining sex linked region (the mutational load hypothesis of Blaser *et al*.^47^). In this type of model, the combined effects of sexually antagonistic mutations and mutational load can potentially lead to repeated turnovers in the locus responsible for sex determination, possibly involving a limited set of chromosomes that carry genes affecting gender development (the “hot-potato model“ of Blaser *et al*.^48^). In these models, the ancestral heterogamety is preserved, as is observed in anurans, though *Glandirana rugosa* (syn. *Rana rugosa*) is an exception^49–51^ and this is supported by the data from other vertebrates reviewed by Blaser *et al*.^48^. (3) Finally, sex ratio selection can play a role if a population’s optimal sex ratio in the population is non-1:1, or if the sex ratio has been distorted by a genomic conflict (reviewed by Beukeboom & Perrin^14^).

As described more fully in the Discussion section, our results allow us to exclude some of these models for *Silene* section *Otites*.

## Results

### Illumina RNAseq sequencing, 454 RNAseq sequencing and genetic analyses

**Figure 2:**
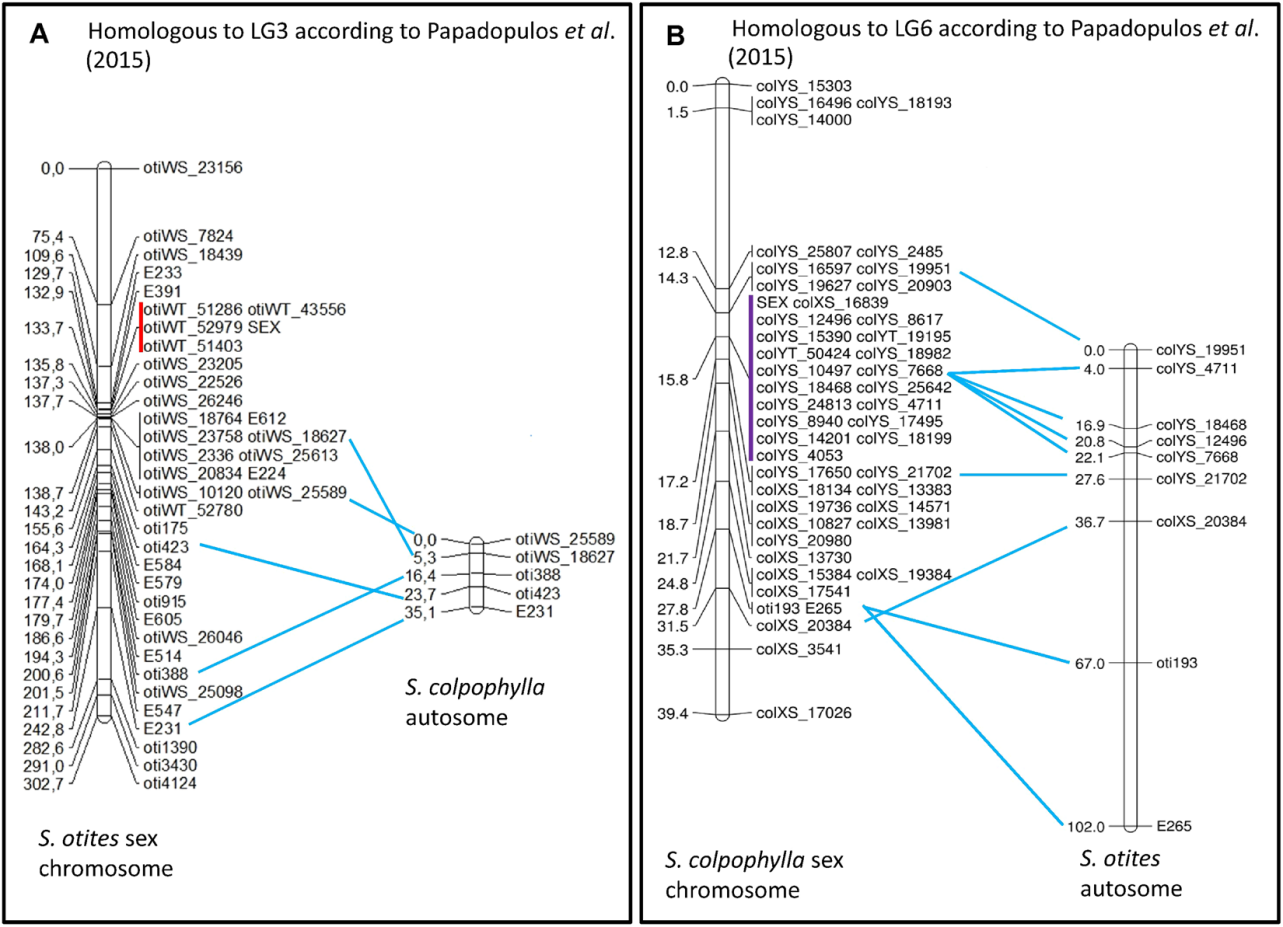
Comparison of the genetic maps of *S*. *otites* and *S*. *colpophylla*. The figure shows that the *S*. *colpophylla* homologs of the sequences that are sex-linked in *S*. *otites* are not sex-linked in *S*. *colpophylla* (**A**) and vice versa (**B**). The completely sex-linked sequences (no recombinants found in the mapping population) that also show complete linkage disequilibrium in natural populations of *S*. *otites* are marked by a red line, and by a violet line in *S*. *colpophylla*.

The overall results of the different approaches employed in this study suggest that sex-determining regions are found on three different chromosomes in the genus *Silene*: two different *S*. *latifolia* autosomes carry homologues of the *S*. *otites* and *S*. *borysthenica* (W) and *S*. *colpophylla* (Y) sex-determining regions, and neither is homologous with the *S*. *latifolia* XY pair (Fig. 2A). We next describe the evidence that supports these conclusions.

#### *S. otites*

Candidate fully or partially sex-linked genes in *S*. *otites* were identified and genetically mapped (Fig. 2A) using several approaches (see Materials and Methods and Supporting information Table S1A). Initial 454 sequencing yielded 36 sequences suitable for genetic mapping in our *S*. *otites* family of 113 plants (see Material and Methods), which identified seven partially sex-linked sequences. These markers are denoted by names beginning with oti, followed by a number. Orthology with these genes identified the *S*. *otites* sex chromosome as the homolog of *S*. *latifolia* linkage group LG6, in the numbering of Bergero *et al*.^52^, or LG3 in the numbering of Papadopulos *et al*.^42^, which we adopt in what follows. Ten further sequences that were previously mapped to this *S*. *latifolia* linkage group^52^ were then identified and genetically mapped in our *S*. *otites* family; these markers’ names begin with E (following Bergero *et al*.^52^). To search for more sex-linked genes, we did Illumina RNAseq using the parents of the same *S*. *otites* family and 6 progeny of each sex. The results were analysed using the SNP based workflow in the LINKYX pipeline^53^ (see Methods), which identified 318 sequences with potentially completely Z-linked and/or W-linked SNPs; these markers are denoted by names beginning with oti, followed by WS (standing for W-linked SNP) and a number. 25 of the sequences identified by the LINKYX pipeline were genetically mapped using the SNP variants, of which 16 were identified as pseudoautosomal and 9 were not localized for technical reasons (unexpected segregation patterns due to multiple copies amplified in PCR). In a third approach, we tested sequences that show female-specific transcription in leaves and flower buds (using LINKYX’s workflow for detecting sex-specific transcripts); this yielded a set of 83 sequences detected only in females in both leaf and flower bud tissues (denoted by names beginning with otiWT, followed by a number (where T indicates that the sequence was detected by analysing transcripts). Further testing using 29 sequences chosen from the set of 83 sequences was done using PCR on genomic DNA in mapping population. In the cases where sex-linked presence/absence PCR polymorphism was not found, we have searched for sex-linked SNP polymorphisms in the corresponding sequences. This study identified one pseudoautosomal sequence (based on mapping an SNP in the mapping population) and four completely sex-linked sequences (based on PCR presence/absence polymorphisms in the mapping population, while the other 24 sequences did not show any sex linkage.

Overall, these three approaches yielded a genetic map with a total of 4 fully sex-linked and 34 pseudoautosomal genes in *S*. *otites*. Fig. 2A shows the genetic map based on all 38 markers (Supporting information Table S1A). The sex-determining locus maps at 138.0 cM, and only the four markers just mentioned showed complete sex linkage; almost the whole of the chromosome’s total map length of 302.7 cM consists of two genetically large pseudo-autosomal regions flanking a fully sex-linked region. All four candidate fully sex-linked polymorphisms showed complete agreement with the sex phenotype in two natural population samples; PCR amplifications of these sequences yielded products only from female genomic DNA samples, and not from males, confirming that they are fully sex-linked (Supporting information Table S2).

To estimate the physical size of the fully sex-linked region, BACs carrying the four fully sex-linked sequences were isolated and sequenced. The W-linked BAC clones proved to have low gene density, consistent with this genome region being non-recombining, and this sequencing revealed only one new sex-linked gene (Supporting information Table S3). The sum of the lengths of the two smallest BACs sequences, plus the smallest distances of the mapped genes from the BAC ends in two another BACs, yields a size of at least 180 kb for the region, yielding a mean of one gene per 36 kb, a very low density.

#### *S. colpophylla*

In *S*. *colpophylla*, the LINKYX (its workflow based on the study of SNPs in RNAseq data) identified 88 sequences with SNPs suggesting either X- and/or Y-linkage. SNPs in 48 of the 88 putatively sex-linked sequences just described were mapped in our sibship of 69 plants. This mapping confirmed full sex-linkage for 16 sequences, and partial sex-linkage for 28 (pseudoautosomal) genes, while 4 were not localized from technical reasons (Supporting information Table S1B). A further 1,671 sequences showed male-limited transcription in flower buds in the same sample of plants (as identified by workflow for detection of sex specific transcripts in LINKYX), but only 22 of these showed male-specific expression in leaves, and only two of them showed male-specific PCR amplification from genomic DNA (one is a sequence with no known homologues, and the other shows homology to F-box proteins). The complete sex linkage of these two sequences was confirmed by mapping of the presence/absence PCR polymorphism in the mapping population. In contrast, only 19 sequences showed female-specific transcription in flower buds, and only one also did so in leaves, and no sequence showed female-specific PCR amplification from genomic DNA. These results support the previous evidence that this species has male heterogamety^38^. In total, we have identified 18 fully sex-linked and 28 pseudoautosomal sequences.

Homologs could be identified between *S*. *otites* or *S*. *colpophylla* for 14 genes showing complete or partial, sex-linkage in one species or the other, and in all cases genes that are sex-linked in one species are autosomal in the other (Fig. 2). The conclusion that the two species have non-homologous sex chromosomes is further supported by screening recently mapped scaffolds from *S*. *latifolia*^42^ with homologs of sex-linked sequences from *S*. *otites* and *S*. *colpophylla*. 29 of the 38 *S*. *otites* sex-linked sequences detected in these scaffolds map to the linkage group identified by our genetic mapping, LG3 (see above), while nine map to five other LGs (Supporting information Fig. S1). 27 homologs of the 29 *S*. *colpophylla* sex-linked sequences detected in these scaffolds map to LG6, and two to LG1 (Supporting information Fig. S1). The discrepancies may reflect incorrect assignments of some *S*. *latifolia* sequences to these scaffolds, or the truly homologous scaffolds were not yet sequenced in some cases and the other ones contained a sequence with sufficient homology to tested sequence as well. Errors in our genetic mapping are much less likely.

Figure 2B shows the genetic map of the *S*. *colpophylla* chromosome carrying the sex-determining locus. The genetic results are consistent with male heterogamety, and our 46 markers place the estimated location of a male-determining locus at 15.8 cM, out of a total map length of 39.4 cM for this chromosome. The fully sex-linked region includes a higher proportion of the sex-linked genes identified than in *S*. *otites*.

The 18 completely sex-linked markers (with no recombinants with the sex-determining locus in our family of 69 progeny) correspond to at a least 16 different genes (in two cases the mapped markers could correspond to different parts of the same gene). However, as markers that were expected to be closely linked to the sex-determining region in *S*. *colpophylla* were preferentially selected for mapping, no reliable comparison can be made of the sizes of the non-recombining region with those in the other species studied here.

#### *S. borysthenica*

For *S*. *borysthenica*, no information on heterogamety was previously available. We again tested sex linkage by LINKYX analysis of SNPs in RNAseq data, using the parents and 12 progeny plants in a full sib family from this species. This approach directly yielded 99 sequences showing probable Z- and/or W-linkage as confirmed in IGV browser. In addition, we tested 318 sequences that showed Z- or W-linked SNP(s) in *S*. *otites*, of which 62 also showed (in IGV browser) complete sex-linkage in this small sample of *S*. *borysthenica* family; conversely, 47 of the 99 sequences with sex-linkage ascertained directly in *S*. *borysthenica* also showed sex-linkage in our RNAseq sequenced samples of *S*. *otites* family. We therefore conclude that the same chromosome carries the sex-determining gene in both these species. 19*S*. *borysthenica* putatively sex-linked sequences showed homology with mapped *S*. *latifolia* scaffolds. Consistent with our identification of the chromosome carrying the *S*. *otites* sex-determining locus, 13 of these (7 newly identified in *S*. *borysthenica* and 6 that are sex-linked both in *S*. *borysthenica* and *S*. *otites*) were detected in LG3 scaffolds; all except one of the scaffolds carrying homologs of the *S*. *otites* and *S*. *borysthenica* sex-linked genes are located in the upper arm of *S*. *latifolia* LG3 (Supporting information Figs S1 and S2). Finally, we tested (using presence/absence PCR polymorphism) sex linkage of the *S*. *borysthenica* gene borW44973, which is the homolog of otiW43556, one of the four *S*. *otites* genes showing complete W linkage and female specific expression; we used a larger mapping population of 32 plants from the same sibship as the used for RNAseqs. As expected under a ZW system, all females but no males had the allele that was assigned as W-linked in our smaller sample of this *S*. *borysthenica* sibship.

### Phylogenetic analyses: Origin of female heterogamety within section *Otites*

Our Bayesian phylogenetic analysis yielded estimated phylogenetic relationships between members of the section *Otites* (see Fig. 1). *S*. *sibirica* appears to be a very close relative of section *Otites* (the estimated time of the most recent common ancestor is about 0.72 MYA). Our phylogenetic analysis estimates that the dioecious section *Otites* evolved very recently, with an age of 0.55 MYA. *Silene sibirica* is not dioecious, but is gynodioecious, having female and hermaphrodite sex morphs. Gynomonoecious individuals with hermaphrodite and female flowers are also occasionally found (Supporting information Fig. S3).

Our BayesTraits analysis infers (see Methods) female heterogamety for both the most recent common ancestor of the section *Otites*, and of the *Otites* group within this section (Supporting information Table S4a,b). MultiState analysis in BayesTraits V3.0 evaluates this scenario as 5 times more probable than the next most probable scenario, constituting a significant difference according to the scale proposed by Kass and Raftery^54^, which considers twofold or greater differences in ln(marginal likelihood) values to be significant. Given the inferred tree, ancestral female heterogamety is consistent with the fact that both the extant species with female heterogamety, *S*. *otites and S*. *borysthenica* (see Fig. 2) have sex-determining loci located on a chromosome homologous with the same *S*. *latifolia* LG (LG3); see Supporting information Fig. S1).

In the case of the two *Otites* group species *S*. *colpophylla* into *S*. *pseudotites*, however, their shared male heterogamety could reflect gene flow from *S*. *colpophylla* into *S*. *pseudotites* (or its ancestor). This possibility is evaluated in the Discussion section. We, therefore, also allowed *S*. *pseudotites* to have either male or female heterogamety. We again calculated the probabilities of all nine possible following scenarios ((i) both section and group *Otites* ZW, (ii) section *Otites* ZW, group *Otites* XY, (iii) section *Otites* ZW, group *Otites* non-dioecious, (iv) section *Otites* XY, group *Otites* ZW, and (v) both section and group *Otites* XY), (vi) section *Otites* XY, group *Otites* non-dioecious), (vii) section *Otites* N, group *Otites* ZW), (viii) section *Otites* non-dioecious, group *Otites* XY) and (ix) both section and group *Otites* non-dioecious. The conclusion that the most recent common ancestor of both the section *Otites* and group *Otites* had female heterogamety is then 17 times more probable than the next alternative. As *S*. *colpophylla* males are heterogametic, this implies that there was a change in heterogamety, as well as a change in the chromosome carrying the sex-linked genes, during the evolution of this species. The chronogram therefore suggests that male heterogamety evolved on the *S*. *colpophylla* branch within group *Otites* about 0.35 MYA (Fig. 1).

## Discussion

Our results confirm the male heterogamety in *S*. *colpophylla* and female heterogamety in *Silene otites* previously inferred using AFLP^17,38^ and markers from 454 sequencing^17^, and demonstrate the presence of a large region with suppressed XY recombination in *S*. *colpophylla* (corresponding to a large homologous region spanning at least 18 cM in *S*. *otites*), and our new data reveal that the chromosome carrying the sex-determining genes in *S*. *colpophylla* evolved from different autosome pair from that carrying the *S*. *otites* sex-determining genes. The overall results (Fig. 1, Supporting information Table S4a,b) suggest that the ancestral state in both section *Otites* and group *Otites* was female heterogamety, and that the original sex-determining region evolved on the homolog of autosomal linkage group 3 of *S*. *latifolia*.

What are possible scenarios for changes in heterogamety in section *Otite*s? The scenario most strongly supported by our phylogenetic analyses suggests that the most recent common ancestors of the whole section *Otites* and of the group *Otites* had female heterogamety (Supporting information Table S4). Combined with the results of our genetic analyses, we can reject a simple scenario involving single origin of dioecy and a single change in heterogamety without any further event such as introgression of a sex chromosome from another species. Four alternatives are possible. The first two scenarios involve male heterogamety as the ancestral state in section *Otites*, while the other two involve ancestral female heterogamety, as the analysis of ancestral states suggests. We next consider the arguments against the first three scenarios, and the evidence that the fourth one is more plausible.

First, even if male heterogamety was ancestral in section *Otites* and female heterogamety evolved independently, it is not parsimonious to propose that the same autosome evolved independently to become a sex chromosome. In the second possibility, male heterogamety is again ancestral in section *Otites* but it changed to female heterogamety in section *Cyri* and a W chromosome introgressed into *S*. *otites*, over-riding its previous sex-determining system, and resulting in female heterogamety in this species. Again, the analysis of ancestral states does not support this. Moreover, introgression is unlikely, given the geographic and phylogenetic distance between species of groups *Otites* and *Cyri*. Species of the *Cyri* group have mostly eastern distributions (in Ukraine and Russia) or are endemic (*S*. *velebitica* in Croatia), whereas *S*. *otites* occupies western and central European localities^55^. Contact in the past cannot, however, be excluded. The third possibility is that, as the analysis of ancestral states suggests, female heterogamety was ancestral in section *Otites* and male heterogamety evolved independently. Again, we argue against this because the hypothesis that the sex chromosomes evolved from the same autosome is not parsimonious.

The final possibility again involves ancestral female heterogamety, as supported by the analysis of ancestral states, and that this changed to male heterogamety in *S*. *colpophylla* and that the Y chromosome from this lineage was introgressed into *S*. *pseudotites*. Although this scenario is also not parsimonious, introgression between these species is likely. There is evidence that hybrids occur naturally between *S*. *colpophylla* and *S*. *pseudotites*, and between *S*. *otites* and *S*. *pseudotites*, in central eastern France, and hybrids between *S*. *colpophylla* and *S*. *otites* are phenotypically similar to *S*. *pseudotites*^55^. Our molecular data, however, suggest that our *S*. *pseudotites* sample from Trieste is not such a hybrid, and suggest that it could be a separate species, sister to *S*. *otites* (we estimate the age of the most recent common ancestor to be > 0.2 MYA). Introgression from *S*. *colpophylla* cannot have occurred recently in Italian populations of *S*. *pseudotites* as *S*. *colpophylla* does not occur in Italy, but it could have occurred in the past. The hybridization hypothesis is further supported by evidence that the sex chromosomes of *S*. *pseudotites* correspond to the *S*. *latifolia* LG6 and male heterogamety in *S. pseudotites* was recently confirmed by molecular methods (unpublished results of the group of Prof. Pascal Touzet, University of Lille 1, France, Prof. Touzet personal communication). If so, these two species probably share a common sex-determining locus, as required under this hypothesis. This scenario predicts that *S*. *colpophylla*’s sex chromosomes should be younger than those of the ZW species. This is not incompatible with our finding that the *S*. *colpophylla* Y chromosome includes a large non-recombining region, as a single large inversion could create such a region^56^, and a large amount of evolutionary time is not required to lead to a large non-recombining region, assuming that the selective situation favouring an inversion has evolved, perhaps involving a sexually antagonistic polymorphism in a partially sex-linked region^57^ (see next section). This inversion hypothesis is empirically testable, because it predicts that *S*. *colpophylla* should have evolutionary strata^58^, but no data are yet available.

An origin of *S*. *colpophylla’s* XY sex-determining system from an ancestral ZW sex-determining system in section *Otites* is also consistent with the phenotypes of interspecific hybrids between *S*. *otites* and *S*. *pseudotites*^16,59^. Hybrids possessing both the W-chromosome and Y-chromosome have male phenotypes, i.e. the Y-linked sex determiner is dominant and epistatic to the previously existing factor. In the future, the study of synonymous site divergence between sequences of Y- versus X-linked genes, and W- versus Z-linked ones should enable us to further characterize the XY systems (in *S*. *colpophylla*, *S*. *pseudotites*) and the ZW systems (in *S*. *borysthenica*, *S*. *wolgensis* and *S*. *otites*). The possibility of change in heterogamety through hybridization is an interesting one that has so far not been considered.

Why might species in section *Otites* have changed heterogamety? All three categories of hypotheses outlined in the introduction require the new sex-determining gene to determine sex even in the presence of the ancestral gene (i.e. to be epistatic to the original sex-determining master gene). Although our results do not identify the mechanism responsible for the switch in heterogamety in section *Otites*, the male determiner of *S*. *pseudotites* is indeed epistatic to the female determiner of *S*. *otites*, as WY hybrid plants show the male phenotype^16^, as expected if female heterogamety was the primary sex-determining system, and the male-determining Y-linked regions of *S*. *pseudotites* and *S*. *colpophylla* represent derived states. As the age of the sex chromosomes in this section is very small, and our results suggest that the non-recombining region of the *S*. *otites* W-chromosome carries few genes, models of type 2 (see Introduction) involving genetic degeneration of this region of the W-chromosome appear improbable.

A further argument against a mutational load model to explain the changes in heterogamety in section *Otites* (in either direction), is that changes in the chromosome on which the sex-determining locus is located are not predicted by this type of model. The extent of genetic degeneration is important because, after such a change, the pre-existing sex-determining chromosome can then become homozygous. If this chromosome carries a degenerated Y- or W-linked region, the change may be prevented due to low fitness or complete inviability of homozygotes of such chromosomes. Changed heterogamety in *Silene* section *Otites* is consistent with our observations suggesting that degeneration is likely to be minor in the ZW species. In such conditions, changed heterogamety is, however, possible under the turnover version of category 2 models (see Introduction) that involves a sexually antagonistic polymorphism^60^. Overall, we conclude that the change to a XY sex-determining system in section *Otites* could have involved either sexually antagonistic selection or neutral processes which allowed spread of a new epistatic sex-determining factor. The same two possibilities could also explain sex determination switches in fish such as *Cichlidae*^21^, which also involve changes in the linkage group carrying the sex-determining region.

A difference in the ages, and the extent of genetic degeneration, can potentially explain the difference between the changes in section *Otites* species, versus maintenance of male heterogamety in species in section *Melandrium* (subgenus *Behenantha*), which have retained XY sex chromosomes with no major changes apart from some translocations, including a reciprocal Y-autosome translocation in *S*. *diclinis*^61^ and expansion of the pseudoautosomal region in *S*. *latifolia*^52^. Species in section *Melandrium* have extensive non-recombining Y-linked regions, carrying large numbers of genes, including anther development genes, some of them essential for male fertility^62–65^. Given its numerous genes, making degeneration expected after sufficient evolutionary time, and the estimated 10 MY time since a fully Y-linked region first evolved, the lethality of YY genotypes (reviewed by Westergaard^62^) is predicted, and there is direct evidence that the non-recombining region of the *S*. *latifolia* Y-chromosome has undergone some degeneration^42,66–68^. Further important differences between sex-determining systems in the section *Melandrium* and that in *S*. *otites* are revealed by the genetic properties of so called “androhermaphrodites” (plants with male and hermaphrodite flowers). Such plants are occasionally found in natural populations of *S*. *dioica*^69^ and North American populations of *S*. *latifolia*^70^. They can also be induced by treatment with hypomethylating agents^71,72^. As expected, given that they are modified males, their progeny include male, females and androhermaphrodites. Phenotypic “androhermaphrodites” were found at an estimated frequency of 0.2% in two natural populations of *S*. *otites* (based on data of Correns^73^). Their progeny included only males and androhermaphrodites, but no females^16,73^, suggesting, in combination with our current data, that their genotype was ZZ, not ZW. An alternative possibility is that these androhermaphrodites do carry W-chromosomes, but that these are abnormal, having compromised female-promoting function, and compromised ability to suppress male functions; in other words, W-chromosomes in some natural populations may be similar to Z-chromosomes, in terms of their sex-determining functions. If so, the female-specific part of the *S*. *otites* W-chromosome cannot be essential for gynoecium formation, i.e. at least some Z-chromosomes carry all genetic information necessary for gynoecium formation.

### Scenario for the evolution of ZW sex determination in section *Otites*

**Figure 3:**
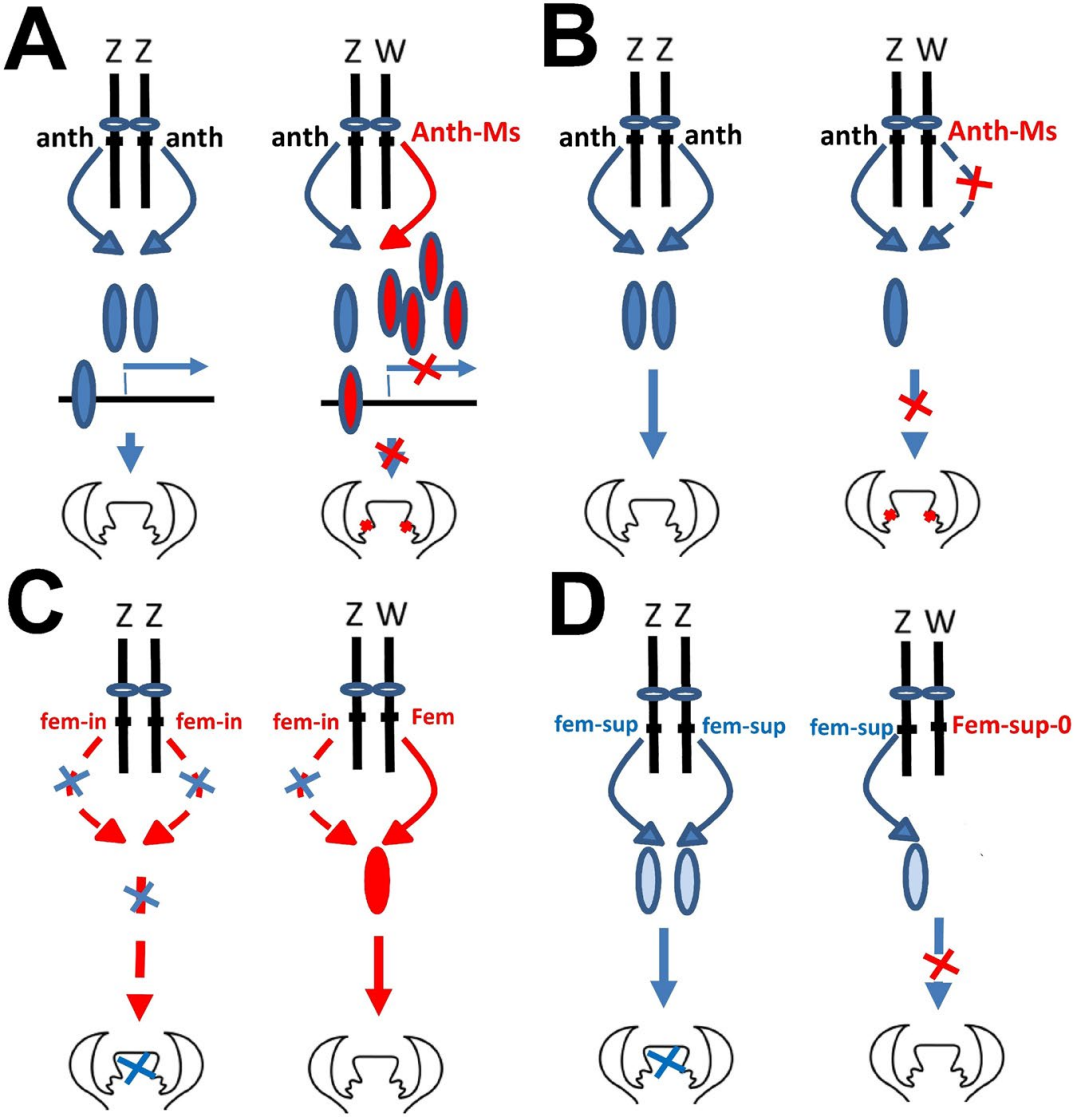
Possible routes to female heterogamety from gynodioecy in the section *Otites*. Possible types of anther suppression mutations on the proto-W-chromosomes (**A**,**B**) and possible mutations causing gynoecium suppression (**C**,**D**). (**A**) A dominant negative W-linked anther suppressor causes male sterility by repressing the transcriptional targets of a Z-linked transcription factor. A W-linked inactive variant of the transcription factor competes with the active variant for its binding site in *S*. *otites*. (**B**) Anther suppression caused by a haploinsufficient loss of function mutation on the proto-W chromosome (**C**) A recessive loss-of-function mutation on the proto-Z chromosome. The presence of androhermaphrodites in *S*. *otites* is difficult to explain under this model. (**D**) Recruitment of dosage-dependent Z-linked gynoecium-suppressing alleles. The W chromosome carries no gynoecium-suppressing alleles. This hypothesis can explain the existence of ZZ androhermaphrodites. Anthers or gynoecium with compromised functions are indicated by red or blue crosses over their primordia.

In speculating about the possible *de novo* evolution of ZW sex determination in section *Otites* species (or in the genus *Fragaria*), a two-locus gynodioecy model is most parsimonious, as the closest relatives (*S*. *sibirica* in section *Otites*) are gynodioecious. If female heterogamety was the first sex-determining system to evolve, male function must have been suppressed by a dominant male-suppressing mutation (rather than a recessive loss-of-function male-sterility mutation as proposed for the evolution of male heterogamety^1^. Such an effect could certainly arise by a dominant negative mutation directly creating a dominant female-determining chromosome (the mutation would define a proto-W-chromosome, see Fig. 3A). This mechanism is similar to the action of the W-linked homologue of the *Xenopus laevis* gene that counteracts *DMRT*1 expression by repressing its transcriptional targets^74^. Alternatively, it might involve a loss-of-function proto-W linked mutation of a haplo-insufficient gene important in anther development, such that proto-Z/proto-W plants, with only a single copy of the functional allele cannot develop normally functioning anthers (Fig. 3B). This mechanism resembles the presumed action of the *DMRT1* gene in the gonadal sex determination in birds (reviewed by Kopp^75^).

To create males, a second mutation is required. A recessive loss-of-function female-sterility mutation (Fig. 3C) cannot readily account for the *S*. *otites* androhermaphrodites, which (as explained above) appear to be ZZ individuals. Therefore, one must invoke a dosage-dependent proto-Z linked allele causing gynoecium suppression when homozygous, but not when present in a single copy in ZW plants (Fig. 3D). As in the two-locus model of the origin of sex determination with male heterogamety (reviewed by Charlesworth^76^), such an allele is more likely to invade the population if it occurs in a locus that is closely linked (in repulsion) to the male-suppressing locus. This mechanism would again be similar to the proposed action of the *DMRT1* gene in birds. In birds, however, *DMRT1*’s female-suppressing effect is indirect, through its role in the formation of male gonad from an originally bipotential tissue (reviewed by Kopp^75^) and so one gene can perform both “male promoting” and “female suppressing” functions.

An interesting aspect of the scenarios outlined here, which assume dosage-dependence, is that the W-chromosome need not contain any sex-determining genes. This would make the ZW sex-determining system prone to become epistatically recessive to the male-determining Y in XY systems. For example, a Y-chromosome might arise through a duplication of a Z-linked gene that promotes male functions in ZZ individuals. Under the model of Veller *et al*.^44^, no special driving force (such as a sexually antagonistic allele) is necessary for a change in sex determination, and the spread of a new dominant male-determiner can occur by drift, potentially explaining the change(s) in section *Otites* within a short time period.

Further progress in understanding of sex determination in the section *Otites* can be achieved by the study of the genes from the non-recombining regions of the species of section *Otites* in this work. In *S*. *otites*, four genes isolated from the non-recombining region represent good candidates for the putative sex-determining genes. OtiWT_51286 shows homology to leucine rich repeat receptor kinase proteins. This large protein family shows various functions, one of its members take part in meristem maintenance (CLV1)^77^. OtiWT_52979 is pentatricopeptide (PPR) repeat-containing protein. This large family of modular RNA-binding proteins is important in regulation of gene expression primarily in organelles but also in the nucleus. Some of them act as fertility restorers^78^. OtiWT_43556 shows homology to zinc-finger motif containing protein CRABS CLAW that is among others involved in the control of the flower meristem termination^79^. OtiWT_51403 codes for putative tetrapeptide repeat homeobox like protein so it is probably a transcription factor^80^ and its role in sex determination cannot be excluded. In further studies of these sequences, it should be kept in mind that the W-linked alleles can act as dominant negatives or they can be just inactive. There are also several putative sex-determining genes among the genes from non-recombining region of *S*. *colpophylla*. The role in the control of male fertility can show colpYS_12469 that is putative LYR protein (leucine/tyrosine/arginine motif containing proteins interacting with mitochondrial protein complexes)^81^. The role in the control of the carpel development (probable gynoecium suppression role in males) is expected in colpYS_7668 and colpYS_25642 showing similarity to CLV1^82^. These two sequences can represent distant part of the same gene. In the case of *S*. *colpophylla*, the sex-determining genes are epistatically dominant to the previous sex-determining system. We therefore expect that the products of Y-linked alleles are biologically active and the future studies of sex determination should be easier in this species.

## Material and Methods

### Plant material and sex systems of the species studied

Information about all the species in our phylogenetic and/or genetic analyses is summarized in the Supporting information Table S5. The male heterogamety in *S*. *pseudotites* and female heterogamety in *S*. *wolgensis* were inferred from results of interspecific crosses^16,59^, and the *S*. *pseudotites* conclusion was recently confirmed by results obtained by the group of Prof. Pascal Touzet, University of Lille 1, Lille, France). The ZW system in *S*. *otites* was previously known^17^, and is confirmed by the present study. *S*. *otites* was used as the second parent in the interspecific crosses whose results are summarized above^16,59^. In all dioecious species studied here, the male and female phenotypes of individual plants are easily distinguishable from the flowers without dissection.

Candidate fully or partially sex-linked genes were identified using several different approaches (see the main text and Supporting information Table S1A,B). In all three species studied, sex linkage was tested in F1 full sibships produced by controlled crosses; the family sizes were 113 (58 females and 55 males) in *S*. *otites*, 32 (15 females and 17 males) in *S*. *borysthenica*, and 69 (27 females and 42 males) in *S*. *colpophylla*. In *S*. *otites*, tests for sequences completely linked to the W-chromosome (showing presence/absence polymorphism in PCR reactions with primers targeting sequences for which preliminary data suggested sex linkage) were done by PCR amplifications in plants sampled from two natural populations from the Czech Republic, located in Rohatec (Hodonin district) (sampled 15 females and 17 males) and Brno-Kralovo Pole (Brno city) (sampled 12 females and 18 males). Presence of a product indicates linkage disequilibrium with a female-determining allele.

### Illumina RNAseq sequencing, Roche 454 RNAseq sequencing and genetic analyses

Initial Roche 454 RNAseq of *S*. *otites*, using cDNA from the seed parent of the F1 sibship used by Slancarova *et al*.^17^ to identify the first sex-linked genes from this species, was performed by GATC Biotech (Constance, Germany). The assembly was performed using mira3 assembler^83^ using default setting. To minimise amplification of paralogs, which would complicate tests for sex linkage, single-copy sequences were chosen from genes in this dataset that showed homology to single-copy nuclear genes in a plant species set that includes species in several genera, *Arabidopsis*, *Populus*, *Vitis* and *Oryza*^84^. Tests for sex linkage, including partial sex linkage, used the families described above, and identified several such genes, identifying pseudoautosomal regions (see below). The linkage group carrying the *S*. *latifolia* homologs of partially or completely sex-linked genes identified in this species was determined using the *S*. *latifolia* genetic map^52^, and proved to be LG6 (in the numbering of Bergero *et al*.^52^). The *S*. *otites* homologs of other genes from this chromosome were then tested for sex linkage in *S*. *otites* and mapped using the same *S*. *otites* sibship.

The main approach used for the identification of sex-linked sequences, and to obtain the sequence data for phylogenetic analysis was Illumina RNAseq (Illumina HiSeq. 2000 PE, 2 × 50 bp and Illumina NextSeq. 500 PE, 2 × 75 bp), performed at the EMBL Genomics Core Facility, Heidelberg (Germany). Sequencing libraries were prepared according to the Illumina protocol using oligo-dT attached magnetic beads. The sequences are deposited in GenBank (https://www.ncbi.nlm.nih.gov/genbank/; see accession numbers in Supporting information Table S6. In *S*. *otites*, *S*. *borysthenica* and *S*. *colpophylla*, the complete list of the sequenced samples including the batch ID, raw read counts, paired read counts after trimming, and mapped and unmapped read counts is summarised in Supporting information Table S7. The evaluation of the quality of Illumina RNA seqs in particular samples has been also performed, to check for possible batch effects see Supporting information Figs S4–S5. Rlog function was used for raw read counts normalization, as implemented in DESeq. 2R package. Adapters and low quality sequences were trimmed using Trimmomatic^85^, and errors corrected using Rcorrector^86^. An RNAseq dataset was assembled separately for each of the species using Trinity (with default setting)^87^. The longest ORFs were identified using Transdecoder and annotated using Trinotate^88^. 1082 orthogroups were used in phylogenetic analyses (see below), and were also annotated using the Blast2go pipeline^89^.

In *S*. *otites*, Illumina RNAseq sequences were obtained separately from both parent plants of the family used for genetic mapping (flower buds and leaves, separately), and from six progeny of each sex (flower buds only). For *S*. *colpophylla*, both flower buds and leaves were sampled from the seed parent of our family, but only leaves were available from the pollen donor. However, leaf and flower bud samples of a “full brother” of this plant were also sequenced, as well as from six progeny of each sex. In *S*. *borysthenica*, sequences were obtained from flower buds of both parents and a sample of their progeny (five females and seven males).

The Illumina RNAseq data were used for preliminary screening for candidate sex-linked genes in *S*. *colpophylla*, *S*. *borysthenica* and *S*. *otites*. Sequences with sex-linked inheritance patterns were first identified using the LINKYX pipeline^53^. This uses reference sequences generated using Trinity^87^ and maps reads to this reference using BWA^90^ (with standard settings). Read duplicates are then removed using Picard’s MarkDuplicates function (http://broadinstitute.github.io/picard/). Subsequently, variants are called. SAMtools^91^ with mpileup set to -uf, and bcftools view used with the following parameters: “-p 0.85 -cgv -”. To identify sequences with sex -linked SNPs, the reference is assembled from reads of the homogametic parent (XX mother or ZZ father), using rules for filtering SNPs as described^53^ (see also Supplementary Methods S1–S3).

The LINKYX pipeline was also used to identify genes with sex-specific expression in *S*. *otites*, and *S*. *colpophylla*. To identify sequences with sex specific expression, separate reference sequences were built using all individuals of each sex. The process is otherwise similar to the detection of sex-specific SNPs. If the contigs are male-specifically expressed represent sequences that are present only in males, the female reads will not map to them. The quantification rules used for the filtering to detect such sequences were as previously described^53^ (see also Supplementary Methods S4).

To detect sequences with SNPs with X-linked inheritance patterns in *S*. *colpophylla*, the pipeline was used without modifications. Modifications were made to reflect female heterogamety, to detect SNPs with Z- or W-linked inheritance patterns in *S*. *otites*, and Y-linked inheritance patterns in species with male heterogamety. In *S*. *borysthenica*, for which no previous genetic information was available, we tested for both male and female heterogamety. The latest version of the pipeline, including all the modifications used here, is available at GitHub repository: https://github.com/biomonika/linkyx. To validate the conclusions from LINKYX, reads from individual plants were mapped to the candidate sex-linked sequences using Bowtie 2^92^ and the output was visually checked in the IGV browser^93^. The examples of the output from this control are shown in Supporting information Fig. S6.

The variants used for genetic mapping included DFLP (DNA fragment length polymorphism)^94^, intron size variants (ISVS, see Bergero *et al*.^40^), and single nucleotide polymorphisms (SNPs). SNPs in our mapping populations were genotyped as CAPS or dCAPS markers^95^, or by a high-resolution melting technique^96,97^. Genetic maps were constructed using JoinMap version 4.1^98^, using the algorithm for a full-sib family in an outbreeder (called CP as “cross pollinators”). The linkage groups were inferred based on LOD scores for independent assortment higher than 4. We used regression mapping and Haldane’s mapping function (the Kosambi mapping function is not supported for CP mapping populations in JoinMap 4.1). Genetic map figures were produced using MapChart^99^. Homologies between the sex chromosomes of *S*. *otites*, *S*. *borysthenica* or *S*. *colpophylla* and *S*. *latifolia* chromosomes were further studied using blastn^100,101^ searches of *S*. *latifolia* scaffolds carrying homologs of genes showing sex linkage in section *Otites* species (with the following parameter values: minimal homology = 75%, minimal length of homologous region = 160 bp, and maximal E-value = 1.e^-35^). For scaffolds that have been genetically mapped in *S*. *latifolia*^42^, we recorded the locations of those carrying the homologs of sex-linked genes from section *Otites* species.

### Phylogenetic analyses, species tree estimation and analysis of ancestral states

Phylogenetic analyses were based on sequences of single-copy genes with orthologues that were found (based on Illumina RNAseqs and transcriptome assemblies) in 10 species of the subgenus *Silene* of the genus *Silene* (we refer to these sequences as orthogroups). We identified 1,082 such orthogroups using Orthofinder^102^, which is based on an MCL algorithm^103^. For the phylogenetic analysis, we mapped the reads from each species to the *S*. *otites* reference sequence using Bowtie 2^92^. We also applied a phasing procedure (to avoid biases in estimates of divergence times, as Lischer *et al*.^104^, have shown that unphased data can produce errors). For individuals without pedigree information, we performed read-based phasing in WhatsHap software^105^, and for samples from *S*. *borysthenica*, *S*. *colpophylla* and *S*. *otites* with pedigree information, we did read-based pedigree phasing^106^, using pedigree phasing mode in WhatsHap^105,106^. Estimated haplotypes were extracted from VCF files using a custom python script and the BCFtools-call method of Samtools v1.3.1^91^. When several alternative phases were found (40 orthogroups), the most parsimonious (the one requiring the fewest substitutions in the data) were chosen. Three out of 40 orthogroups were discarded due to phasing ambiguity (more than one equally parsimonious phase). Low coverage sites with a read depth below 2 were masked using the BEDtools^107^ maskfasta method. Multiple sequence alignments were performed using the FFT-NS-i option^108^ of MAFFT v7.13^109^.

In order to conduct a fully parameterized multiple coalescent analysis, only *S*. *sibirica*, the closest non-dioecious species, was used as the outgroup. To avoid possible paralogous sequences, all alignments with more than two alleles in the single individual representing any of the taxa were removed before further analyses. All remaining alignments were manually checked for phasing ambiguities, and branch lengths appearing excessively long were excluded after visual inspection. This affected fourteen orthogroup alignments (2% of the dataset after removal of alignments with more than two alleles). In eight of these, the long branches appeared likely to be due to paralogy, as the aberrant allele formed a separate branch from the other alleles, but the species topology was the same in both branches (”mirror image topologies”**)**. The final phased data set consisted of 662 alignments.

The data were analyzed using the Starbeast2 version 0.13 plugin^110^ and BEAST2^111^ version 2.4.7. We used a strict clock and a Jukes-Cantor substitution model for all alignments. A lognormal distribution with parameters (4.6, 2) was used for the speciation rate, and, for the population size, a lognormal distribution (−7, 2) was used as prior. The Markov Chain Monte Carlo was run for 1,000,000,000 iterations. For the time estimates, we used calibration based on estimated divergence per synonymous site *dS* (substitutions per synonymous site) and the divergence time estimates for several angiosperm lineages (*Brassicaceae*, *Malvaceae*, *Euphorbiaceae*, *Fabaceae*, *Cucurbitaceae*, *Rosaceae*, *Solanaceae*, and *Poaceae*), so as not to depend on a single fossil record or phylogenetic tree position, which yielded a mean substitution rate of 5.35 × 10^−9^ synonymous substitutions/site/year^112^. Mean *dS* values between the *S*. *sibirica* outgroup species and each section *Otites* species were estimated for each gene using the HyPhy package^113^. The mean *dS* is 0.0077; using the above angiosperm substitution rate per year, and assuming one generation per year for the *Silene* species, the estimated age of the most recent common ancestor of the *S*. *sibirica* and the section *Otites* species is about 720 thousand years (SE = 13 thousand years).

Ancestral state analysis was performed in BayesTraits V3.0 using the MultiState method^114^. The analysis took into account all currently available information about male and female heterogamety in the section *Otites*, including previously published phenotypic data^16,59^. All nine possible combinations of ancestral states of the most recent common ancestor of the whole section *Otites* and of the group Otites (see Supporting information Table S4a,b) were tested ((i) section *Otites* ZW, group *Otites* ZW, (ii) section *Otites* ZW, group *Otites* XY, (iii) section *Otites* ZW, group *Otites* N, (iv) section *Otites* XY, group *Otites* ZW, and (v) section *Otites* XY, group *Otites* XY), (vi) section *Otites* XY, group *Otites* N), (vii) section *Otites* N, group *Otites* ZW), (viii) section *Otites* N, group *Otites* XY) and (ix) section *Otites* N, group *Otites* N; female heterogamety-ZW, male heterogamety –XY, non-dioecious-N). As the input we used the set of the species trees obtained using the Starbeast2 plugin^110^ and BEAST2^111^. The chains were run for 10^9^ states, using a gamma hyper-prior (mean from uniform: 0–10, variance from uniform: 0–10). Again, the process was checked using Tracer^115^. The marginal likelihoods for the Bayes factors were obtained using the stepping-stones method following the BayesTraits V3.0 tutorial. The significance of differences was evaluated according to Kass and Raftery^54^.

### BAC library construction, screening and the subsequent analyses

A partial genomic BAC library was constructed for *Silene otites* (sample NN1 from the Nantes (France) population) using high molecular weight DNA prepared from flow-sorted nuclei. The protocol was modified from Cegan *et al*.^116^, for the details see Supporting information Methods S5. Library screening was performed by radioactive hybridization with ^α^32P using the Prime-It II Random Primer Labelling Kit (Stratagene) following Cegan *et al*.^117^. Probes were prepared by PCR amplification of selected markers. Positively hybridizing BAC clones were selected, and the presence of a gene of interest was confirmed by PCR and sequencing. BAC DNA was isolated from the positive BACs and sequenced using an Illumina MiSeq (2 × 300PE) machine at the Centre of Plant Structural and Functional Genomics (Olomouc, Czech Republic). The reads were assembled *de novo* using the Edena assembler^118^.

## Supplementary information

Supporting information

## Data Availability

The Illumina RNAseq raw data and Roche 454 RNAseq raw data have been deposited in GenBank (https://www.ncbi.nlm.nih.gov/genbank/; see accession numbers in Supporting information Table S6).
