# Supplementary material for: Evolution of sex determination and heterogamety changes in section Otites of the genus Silene
Source: Sci Rep. 2019 Jan 31;9:1045. doi: 10.1038/s41598-018-37412-x (PMC6355844; doi:10.1038/s41598-018-37412-x)
Supplement: Supplementary file 1 — Supporting information [file 41598_2018_37412_MOESM1_ESM.pdf]

# *Scientific Reports* - Supporting Information

**Article title:** Evolution of sex determination and heterogamety changes in section *Otites* of the genus *Silene*

**Authors:** Veronika Balounova, Roman Gogela, Radim Cegan, Patrik Cangren, Jitka Zluvova, Jan Safar, Viera Kovacova, Roberta Bergero, Roman Hobza, Boris Vyskot, Bengt Oxelman, Deborah Charlesworth and Bohuslav Janousek

**The following Supporting Information is available for this article:**

**Methods S1:** Segregation patterns of Y-linked SNP (male heterogamety) and corresponding filtering rules used in LINKYX
**Methods S2:** Segregation patterns of W-linked SNP (female heterogamety) and corresponding filtering rules used in LINKYX
**Methods S3:** Segregation patterns of Z-linked SNP (female heterogamety) and corresponding filtering rules used in LINKYX
**Methods S4:** Filtering rules for the identification of the contigs with sex specific transcription
**Methods S5:** BAC library construction

**Fig. S1:** Comparison of the location of the sex-linked markers in the three species of the section *Otites* with the location of their *S. latifolia* homologues
**Fig. S2:** Comparison of the mapping positions of the *S. latifolia* homologs of candidate sex-linked markers in *S. otites* and *S. borysthenica*
**Fig. S3:** Sexual phenotypes in *Silene sibirica*
**Fig. S4:** Dendrograms of the Illumina RNAseq samples in *S. otites*, *S. borysthenic*a and *S. colpophylla* based on the clustering (Euclidean distance measure, UPGMA agglomeration)
**Fig. S5:** Analysis of the Illumina RNAseq samples in *S. otites*, *S. borysthenica* and *S. colpophyla* using principal component analysis (rlog normalized counts)
**Fig. S6:** Examples of the output of the control of the sex-linkage of the candidate SNPs identified in LINKYX (both examples show *S. borysthenica* samples

**Table S1A:** List of genetically mapped markers in *S. otites*
**Table S1B:** List of genetically mapped markers in *S. colpophylla*
**Table S2:** Analysis of the linkage disequilibrium at sexual phenotype and four tested loci.
**Table S3:** Overview of the analysis of the completely sex-linked sequences and their surroundings in *S. otites*
**Table S4A:** Analysis of combinations of the ancestral states of sex determining system in the most recent common ancestors of the section *Otites* and in the MRCA of the group *Otites* in BayesTraits (*S. pseudotites* is set as Y)
**Table S4B:** Analysis of combinations of the ancestral states of sex determining system in the most recent common ancestors of the section *Otites* and in the MRCA of the group *Otites* in BayesTraits (*S. pseudotites* is set as WY)
**Table S5:** Overview of the plant species and samples used in analyses
**Table S6:** List of the accession numbers of the NCBI BioProjects storing the RNAseq data
**Table S7:** Overview of the Illumina RNAseq sequenced samples studied by LINKYX

# Methods S1: Segregation patterns of Y-linked SNP (male heterogamety) and corresponding filtering rules used in LINKYX.

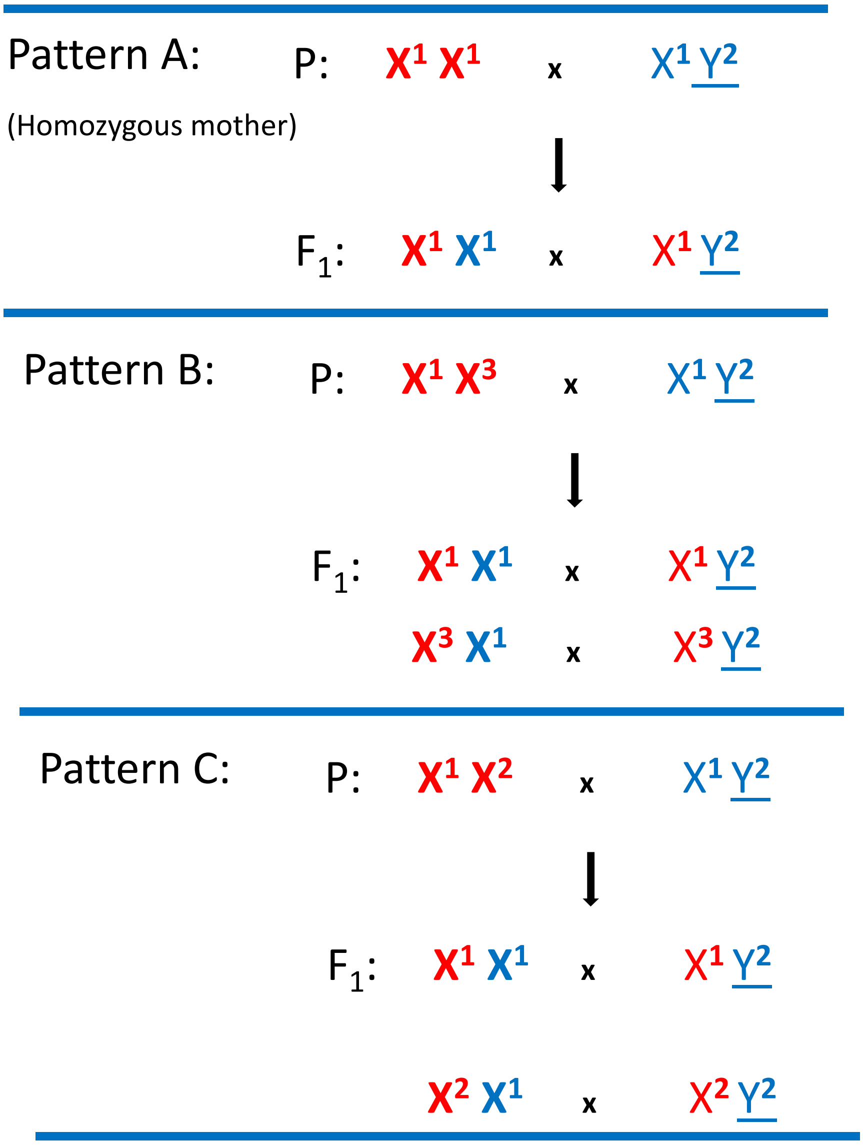


**Filtering rules for identification of Y-linked genes following segregation pattern A:**

1) **Mother must be homozygous ($X^1X^1$) -** Tolerance according to depth of coverage:
   0-4 reads: 0,
   5-24 reads: 1,
   >24 reads: <=4 %
2) **Sons must be same as father ($X^1Y^2$)**
3) **Father must differ from mother (presence of extra allele $X^2$).**
4) **Sons must have mother and father alleles ($X^1Y^2$)**
5) **No "recombinant" individuals are allowed -** Tolerance according to depth of coverage:
   0-4 reads: 0,
   5-24 reads: 1,
   >24 reads: <=4 %.
6) **Sufficient coverage.** Average depth per nucleotide must be at least 0.75 (=in contig of length 100, there must be at least 75 nucleotides present/mapped).

# Methods S2: Segregation patterns of W-linked SNP (female heterogamety) and corresponding filtering rules used in LINKYX.

**Pattern A:**
(Homozygous father)

P: $Z^1 W^2$ x $Z^1 Z^1$

↓

$F_1$: $Z^1 W^2$ + $Z^1 Z^1$

**Pattern B:**

P: $Z^1 W^2$ x $Z^1 Z^3$

↓

$F_1$: $Z^1 W^2$ + $Z^1 Z^1$

$Z^3 W^2$ $Z^1 Z^3$

**Pattern C:**

P: $Z^1 W^2$ x $Z^1 Z^2$

↓

$F_1$: $Z^1 W^2$ + $Z^1 Z^1$

$Z^2 W^2$ $Z^1 Z^2$

## Filtering rules for identification of W-linked genes following segregation pattern A:

1) **Father must be homozygous ($Z^1Z^1$) -** Tolerance according to depth of coverage:
   0-4 reads: 0,
   5-24 reads: 1,
   >24 reads: <=4 %
2) **Daugthers must be same as mother ($Z^1W^2$)**
3) **Mother must differ from father (presence of extra allele $W^2$).**
4) **Daugthers must have mother and father alleles ($Z^1W^2$)**
5) **No "recombinant" individuals are allowed -** Tolerance according to depth of coverage:
   0-4 reads: 0,
   5-24 reads: 1,
   >24 reads: <=4 %.
6) **Sufficient coverage.** Average depth per nucleotide must be at least 0.75 (=in contig of length 100, there must be at least 75 nucleotides present/mapped).

# Methods S3: Segregation patterns of Z-linked SNP (female heterogamety) and corresponding filtering rules used in LINKYX.

Pattern A: P: $\underline{Z^2}\,W^1$ x $Z^1\text{-}Z^1$

(Homozygous father)

↓

$F_1$: $Z^1\,W^1$ + $\underline{Z^2}\,Z^1$

Pattern B: P: $\underline{Z^2}\,W^1$ x $Z^1\text{-}Z^3$

↓

$F_1$: $Z^1\,W^1$ + $\underline{Z^2}\,Z^1$

$Z^3\,W^1$ $\underline{Z^2}\,Z^3$

Pattern B: P: $\underline{Z^2}\,W^1$ x $Z^1\text{-}Z^2$

↓

$F_1$: $Z^1\,W^1$ + $\underline{Z^2}\,Z^1$

$Z^2\,W^1$ $\underline{Z^2}\,Z^2$

## Filtering rules for identification of Z-linked genes following segregation pattern A:

1) **Father must be homozygous ($Z^1Z^1$) -** Tolerance according to depth of coverage:
   0-4 reads: 0,
   5-24 reads: 1,
   >24 reads: <=4 %
2) **Sons must be same as mother ($Z^2Z^1$)**
3) **Mother must differ from father (presence of extra allele $Z^2$).**
4) **Sons must have mother and father alleles ($Z^1Z^2$)**
5) **No "recombinant" individuals are allowed -** Tolerance according to depth of coverage:
   0-4 reads: 0,
   5-24 reads: 1,
   >24 reads: <=4 %.
6) **Sufficient coverage.** Average depth per nucleotide must be at least 0.75 (=in contig of length 100, there must be at least 75 nucleotides present/mapped).

## Methods S4: Filtering rules for the identification of the contigs with sex specific transcription.

**I. Filtering rules for identification of male specific genes**

1) Minimal and maximal number of reads: Each male must have at least 15 reads mapped, each female can have at most 3 reads mapped. Reference is based on reads from all males. Before applying filtering rules, read numbers are normalized to the number of mother reads.
2) Fraction of female and male reads: female reads/(female reads + male reads) < 0.03.
3) Male reads must be more abundant than female reads.
4) mother reads < father reads;
5) mother reads < son reads;
6) daughter reads < father reads;
7) daughter reads < son reads

**II. Filtering rules for identification of female specific genes**

1) Minimal and maximal number of reads: Each female must have at least 15 reads mapped, each male can have at most 3 reads mapped. Reference is based on reads from all females. Before applying filtering rules, read numbers are normalized to the number of father reads.
2) Fraction of male and female reads: male reads/(female reads + male reads) < 0.03.
3) Female reads must be more abundant than male reads.
4) father reads < mother reads;
5) father reads < daughter reads;
6) son reads < mother reads;
7) son reads < daughter reads

# Methods S5: BAC library construction.

Briefly, 25g of *S. otites* fresh leaf tissue was formaldehyde-fixed and homogenized. The crude suspension of intact nuclei was purified by flow-sorting (FACSAria, Becton Dickinson) and embedded into agarose plugs, each averaging 200,000 nuclei. 11 plugs were partially digested with *Hin*dIII enzyme. The digested DNA was separated by pulsed field gel electrophoresis in two size-selection steps, yielding 100-300 kb fragments which were then isolated from the gel and ligated into a dephosphorylated vector pAGIBAC5. The recombinant BACs were transformed into the *E. coli* strain MegaX DH10B T1 (Invitrogen) by electroporation. The library consists of 73,728 clones, which were picked by an automatic robotic station Q-BOT and ordered into 192 384-well microtitre plates. The average insert size was 118 kb, and the library therefore represents 1.5 haploid genome equivalents of *S. otites* (whose genome size is 5.8Gb). Clones were gridded in duplicate onto Hybond N+ (Amersham, Biosciences) nitrocellulose membrane filters following a 464 pattern that allowed us to identify the position and plate number of each clone. Contamination with chloroplast DNA and mitochondrial DNA were negligible, as the nuclei had been purified by flow cytometry.

**Fig. S1: Comparison of the location of the sex-linked markers in the three species of the section *Otites* with the location of their *S. latifolia* homologues** (*S. latifolia* linkage groups are numbered according to Papadopulos et al., 2015; the numbers after the colons show number of markers corresponding to given linkage group).

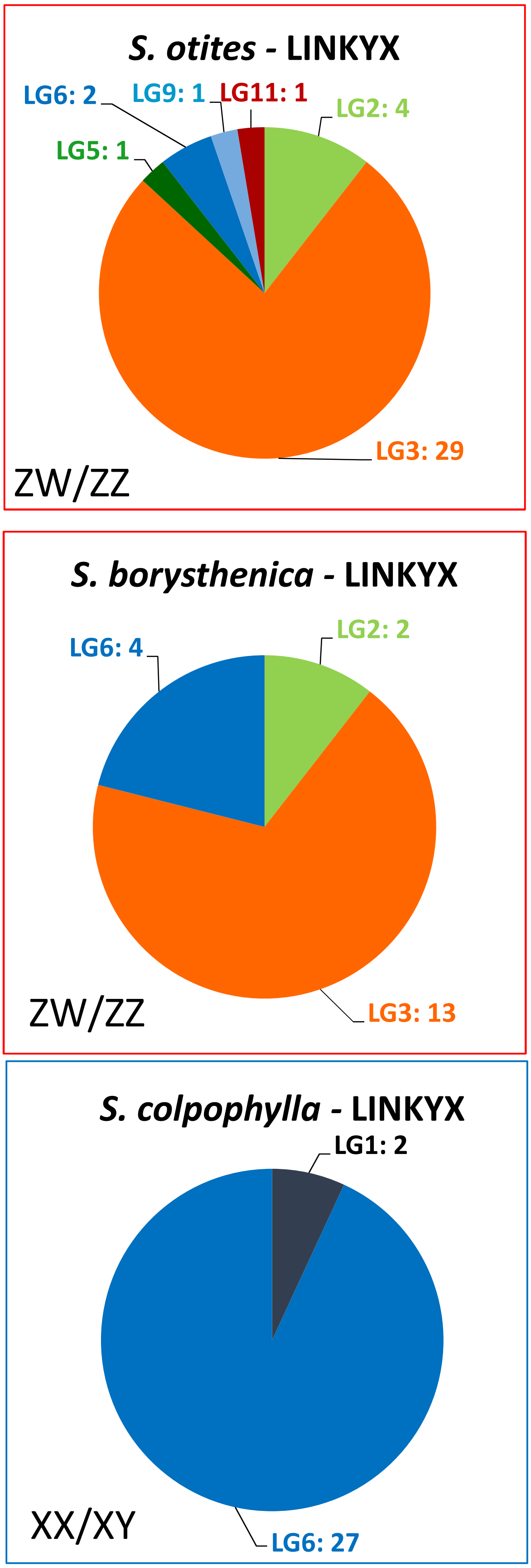

**Fig. S2: Comparison of the mapping positions of the *S. latifolia* homologs of candidate sex-linked markers in S. otites and S. borysthenica.** (LG3 is shown only.)

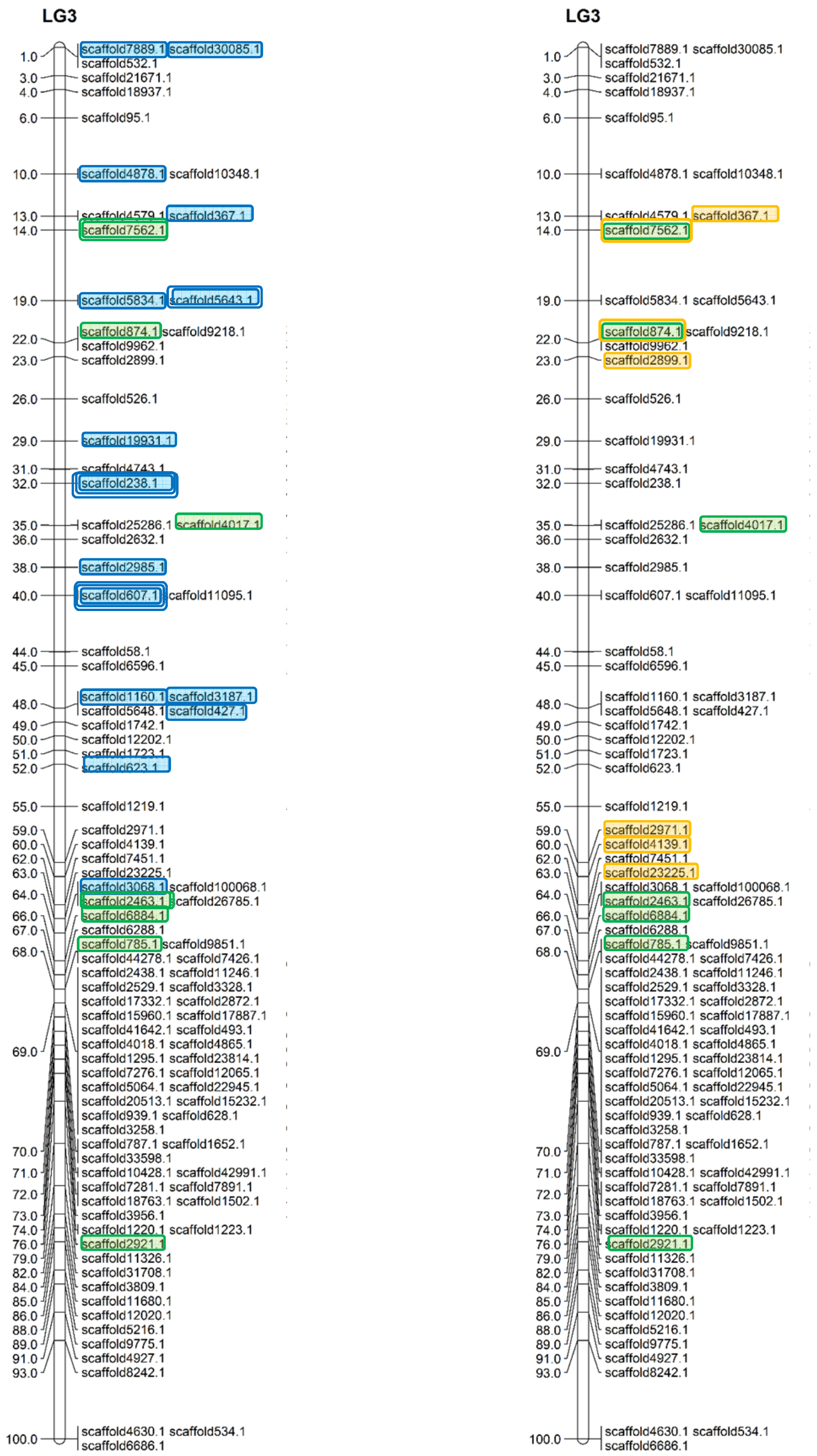


homology with
*S. otites*
candidate sequences

homology with
*S. borysthenica*
candidate sequences

Note: The number of the marked scaffolds is lower than the number of the assigned markers as some of the scaffolds contained homologs of two or three markers (marked by double or triple line). The markers found in both species are marked in green. The markers found in *S. otites* are marked in blue. The markers found in *S. borysthenica* are marked in orange.

**Fig. S3: Sexual phenotypes in *Silene sibirica*.** A - hermaphrodite, B – female, C - gynomonoecious plant, D – details of the female flower in the gynomonoecious plant

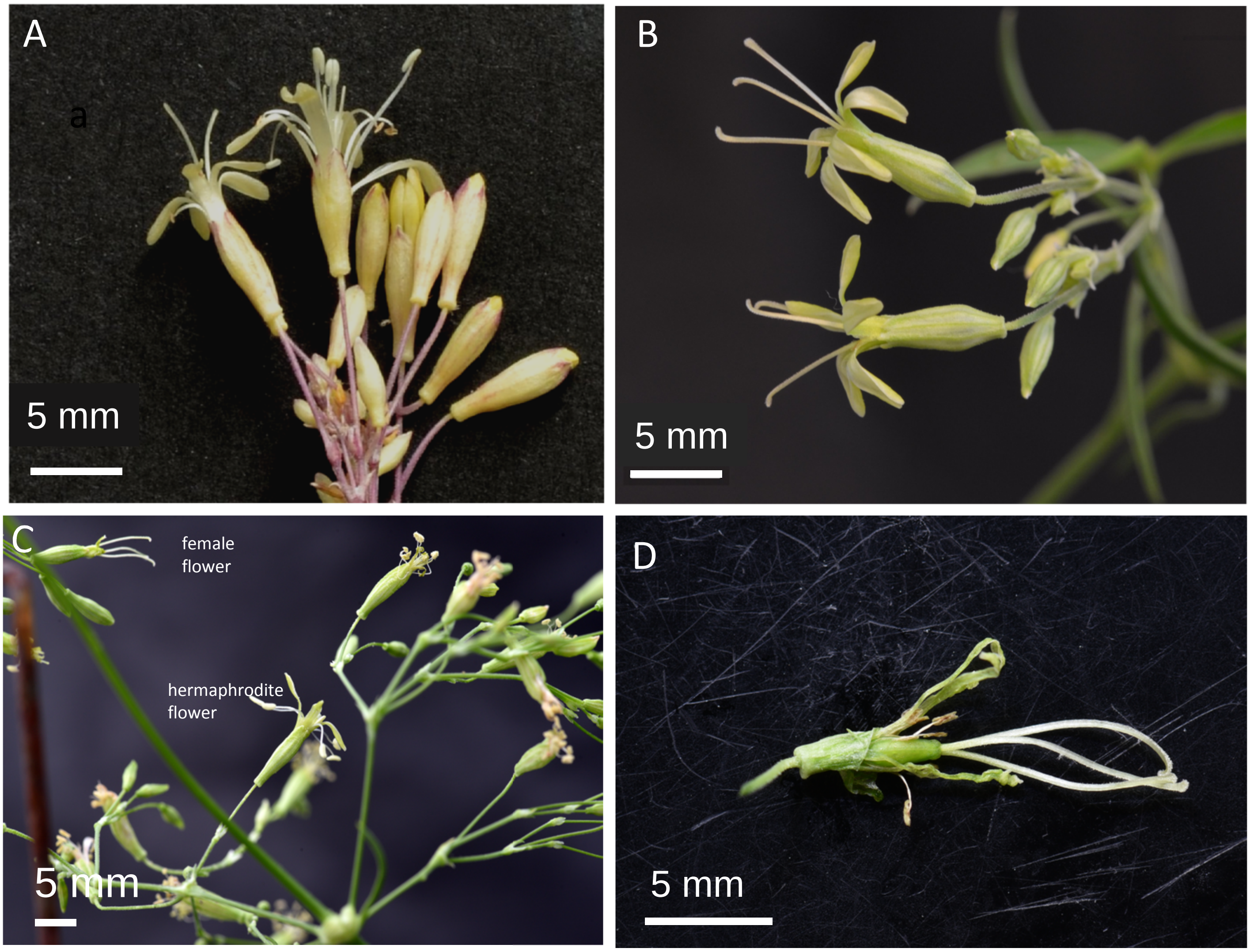

**Fig. S4: Dendrograms of the Illumina RNAseq samples in *S. otites, S. borysthenica* and *S. colpophyla* based on the clustering (Euclidean distance measure, UPGMA agglomeration )**

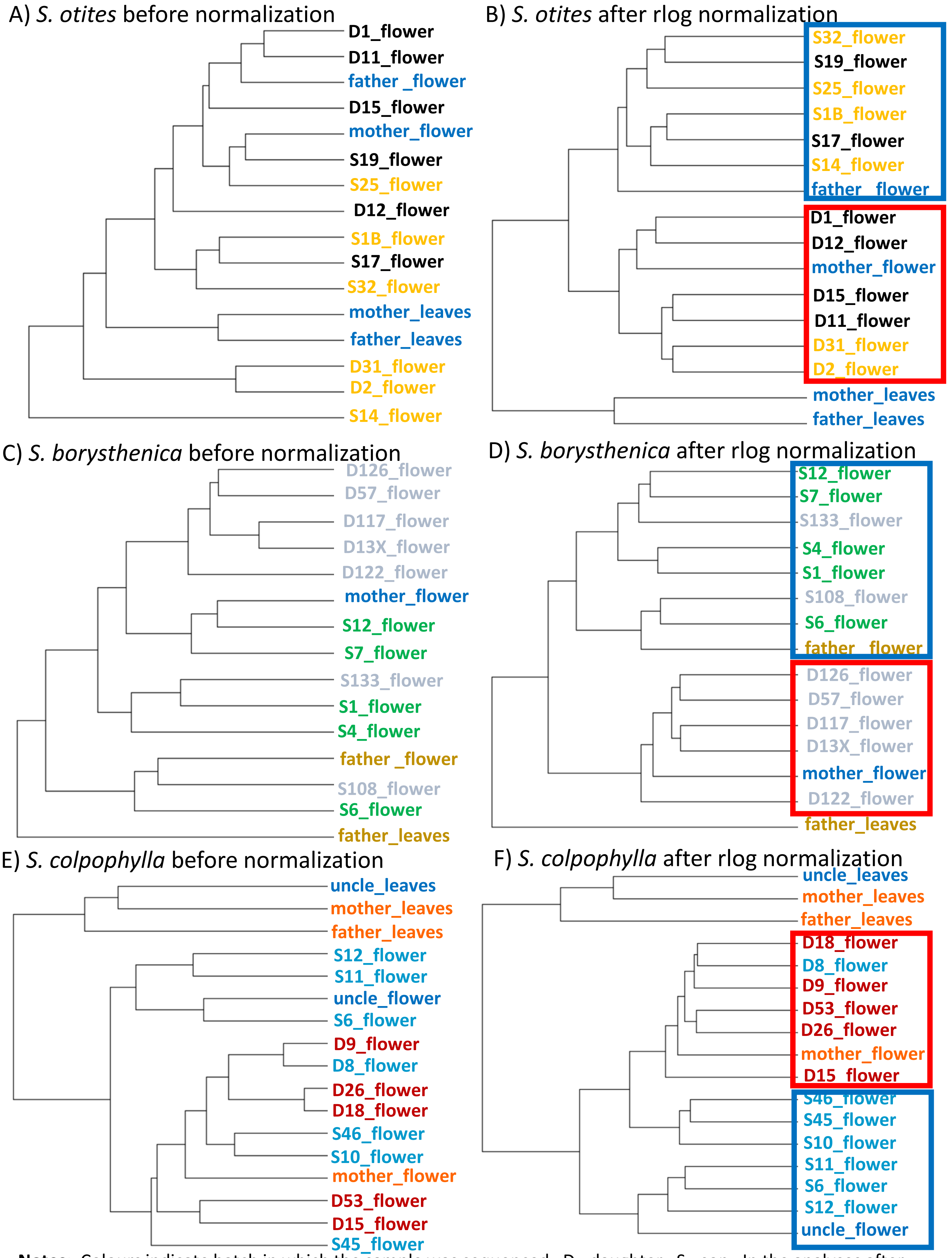


**Notes:** Colours indicate batch in which the sample was sequenced. D - daughter. S - son. In the analyses after normalizaton, the male flower bud samples are marked by a blue and the female flower bud samples by a red frame.

**Fig. S5: Analysis of the Illumina RNAseq samples in *S. otites, S. borysthenica* and *S. colpophyla* using principal component analysis (rlog normalized counts)**

**A) *S. otites*: Batch effect visualisation**

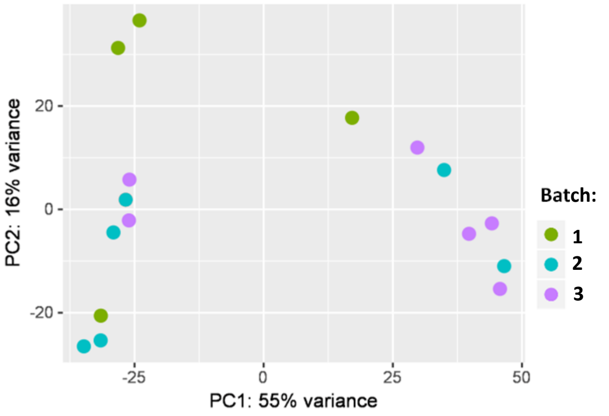


**B) *S. otites*: Effects of the sample type**

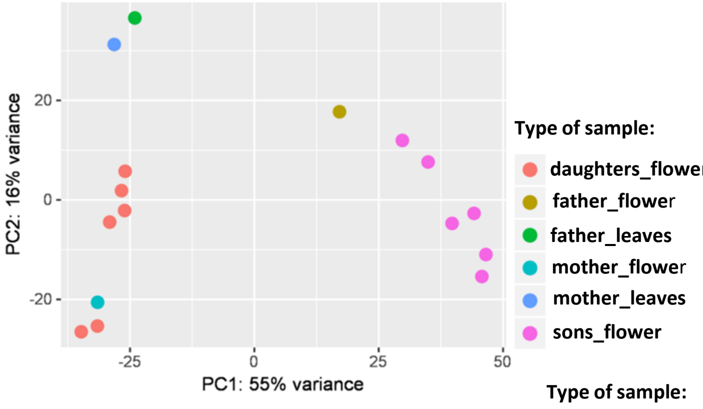


**C) *S. borysthenica*: Batch effect visualisation**

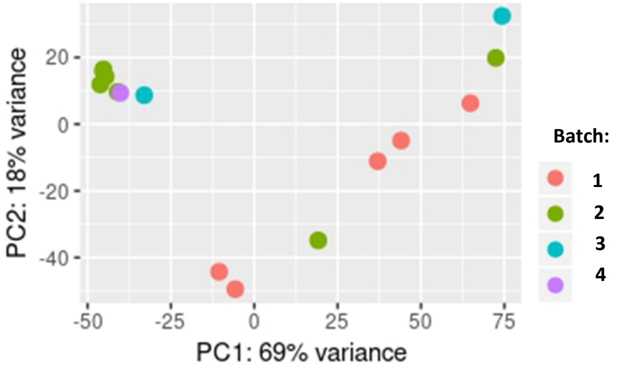


**D) *S. borysthenica*: Effects of the sample type**

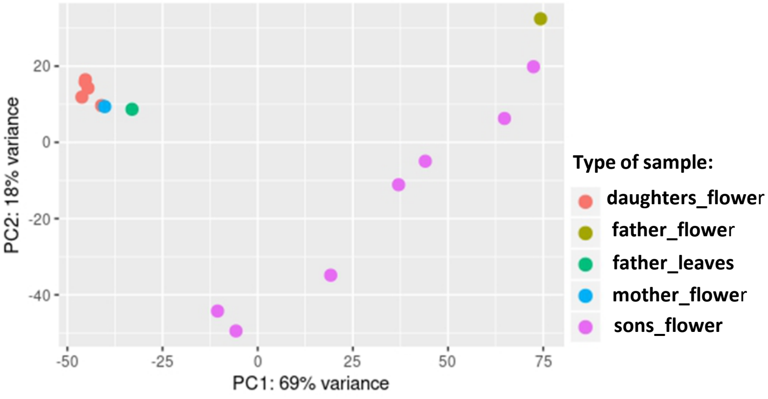


**E) *S. colpophylla*: Batch effect visualisation**

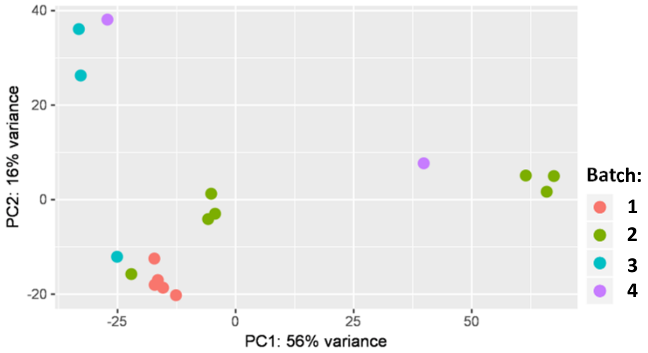


**F) *S. colpophylla*: Effects of the sample type**

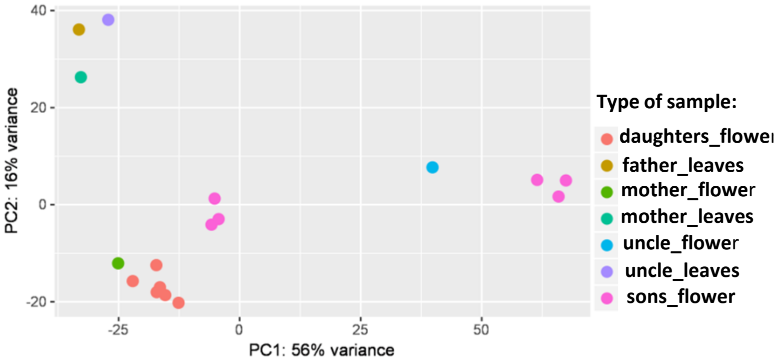


**Comments:** From the comparison of the graphs coloured according to batches with those coloured according to the sample types, it is apparent that the samples are clustered according to the sample type but not according to the batch in which they were sequenced. The female flower bud samples (marked as flower) show more compact clusters then male flower buds. This phenomenon is probably caused by a larger mass of quickly differentiating tissues in male flower buds. In *S. otites* and *S. colpophylla,* the male and female leaf samples (leaves) are always clustering together (in accordance with a very low number of sex specificaly expressed sequences in leaves that was found). In *S. borysthenica,* only father leaf sample was available. The LINKYX search for the sex specifically transcribed sequences was not carried out in this species.

**Fig. S6: Examples of the output of the control of the sex-linkage of the candidate SNPs identified in LINKYX** (both examples show *S. borysthenica* samples)

**A) Analysis of SNPs in the sequence borWS_29170 using Bowtie 2 mapped reads and IGV browser.**

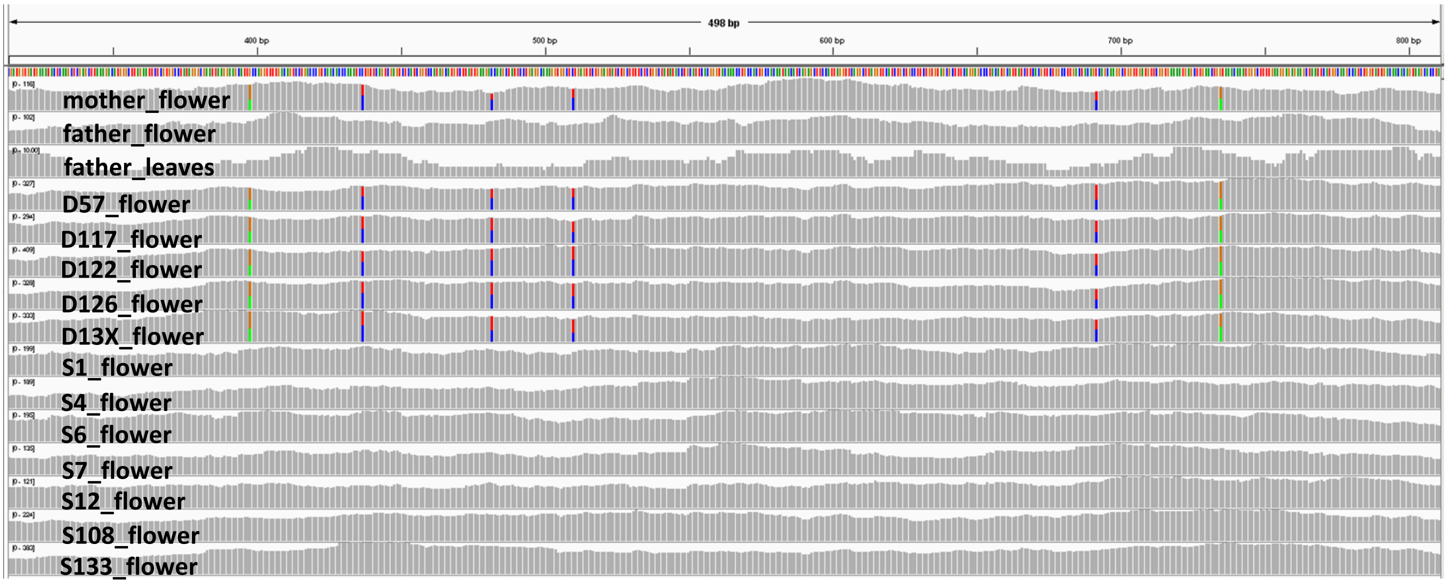


**Comments:** D - daughter. S - son, flower - flower buds. Figure shows 6 co-segregating W-linked polymorphisms. Heterozygous sites occur only mother and daughters.

**B) Analysis of SNPs in the sequence borZS_17684 using the Bowtie 2 mapped reads and IGV browser**

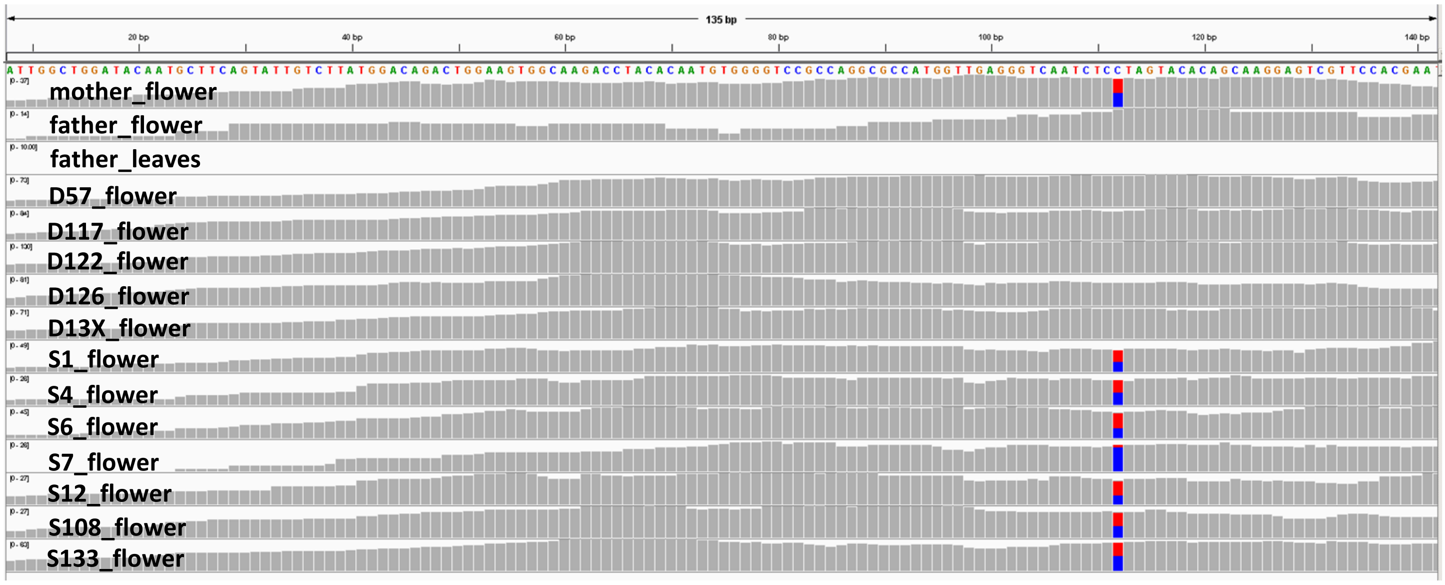


**Comments:** D - daughter, S - son, flower - flower buds. Figure shows one Z-linked polymorphism. Heterozygous sites occur only mother and sons. No reads present in the leaf sample obtained in father are probably caused by tissue specific expression of this sequence.

# Table S1A: List of genetically mapped markers in *S. otites.*

| Locus | origin of markers | homology with |
|---|---|---|
| **otiWT43556** | Illumina RNAseq marker | CRABS CLAW; zinc-finger motif containing protein |
| **otiWT51286** | Illumina RNAseq marker | leucine rich repeat receptor kinase protein |
| **otiWT51403** | Illumina RNAseq marker | tetrapeptide repeat homeobox like protein |
| **otiWT52979** | Illumina RNAseq marker | pentatricopeptide (PRR) repeat-containing protein |
| E224 | homologues from S. latifolia | PREDICTED: 3-isopropylmalate dehydratase large subunit |
| E231 | homologues from S. latifolia | uncharacterized protein |
| E233 | homologues from S. latifolia | PREDICTED: alcohol dehydrogenase |
| E391 | homologues from S. latifolia | PREDICTED: thiamine thiazole synthase 2 |
| E514 | homologues from S. latifolia | PREDICTED: peptidyl-prolyl cis-trans isomerase CYP37 |
| E547 | homologues from S. latifolia | PREDICTED: E3 ubiquitin-protein ligase SP1 |
| E579 | homologues from S. latifolia | PREDICTED: mRNA-decapping enzyme-like protein |
| E584 | homologues from S. latifolia | uncharacterized protein |
| E605 | homologues from S. latifolia | PREDICTED: argininosuccinate lyase |
| E612 | homologues from S. latifolia | PREDICTED: reactive Intermediate Deaminase A |
| oti1390 | 454 sequencing marker | PREDICTED: vacuolar protein sorting-associated protein 18 homolog |
| oti175 | 454 sequencing marker | PREDICTED: chaperonin-like RbcX protein 2 |
| oti3430 | 454 sequencing marker | hypothetical protein |
| oti388 | 454 sequencing marker | Pentatricopeptide repeat-containing protein |
| oti4124 | 454 sequencing marker | PREDICTED: rhodanese-like domain-containing protein 9 |
| oti423 | 454 sequencing marker | uncharacterized protein |
| oti915 | 454 sequencing marker | PREDICTED: tryptophan synthase beta chain 1 |
| otiWT52780 | Illumina RNAseq marker | no homology |
| otiWS_10120 | Illumina RNAseq marker | PREDICTED: inactive protein RESTRICTED TEV MOVEMENT 2 |
| otiWS_18764 | Illumina RNAseq marker | PREDICTED: protein SPA1-RELATED |
| otiWS_22526 | Illumina RNAseq marker | PREDICTED: probable galacturonosyltransferase 12 |
| otiWS_23156 | Illumina RNAseq marker | uncharacterized protein |
| otiWS_23205 | Illumina RNAseq marker | PREDICTED: HVA22-like protein f |
| otiWS_23758 | Illumina RNAseq marker | PREDICTED: pentatricopeptide repeat-containing protein At5g18390 |
| otiWS_25098 | Illumina RNAseq marker | uncharacterized protein |
| otiWS_25589 | Illumina RNAseq marker | no homology |
| otiWS_25613 | Illumina RNAseq marker | PREDICTED: cell cycle checkpoint control protein RAD9A |
| otiWS_18439 | Illumina RNAseq marker | PREDICTED: transcription factor bHLH104 |
| otiWS_18627 | Illumina RNAseq marker | uncharacterized protein |
| otiWS_20834 | Illumina RNAseq marker | PREDICTED: RNA-binding protein 25 |
| otiWS_2336 | Illumina RNAseq marker | PREDICTED: probable glucan 1,3-alpha-glucosidase |
| otiWS_26046 | Illumina RNAseq marker | RNase_T domain-containing protein |
| otiWS_26246 | Illumina RNAseq marker | PREDICTED: F-actin-capping protein subunit alpha |
| otiWS_7824 | Illumina RNAseq marker | uncharacterized protein |
| | | |

Note: The completely sex linked sequences are in bold.
Labelling of markers:
OtiWT- markers found based on their female specific transcription (detected using LINKYX in Illumina RNAseq data). All four completely sex linked markers in the map show also female specific PCR amplification.
OtiWS - markers found based on the W-linked SNP-polymorphism (detected using LINKYX in Illumina RNAseq data).
oti - markers found based on 454 RNAseq data.
E – markers found based on the homology of the ZW chromosome pair with the linkage group 6 in *S. latifolia* in the numbering of Bergero *et al*. (2013) (LG3 in numbering according to Papadopulos et al., 2015). Each this marker corresponds to the homologue in *S. latifolia* with the same label.

# Table S1B: List of genetically mapped markers in *S. colpophylla.*

| Locus | origin of markers | homology with |
|---|---|---|
| **colpXS_16839** | Illumina RNAseq marker | PREDICTED: U-box domain-containing protein 44 |
| **colpYS_10497** | Illumina RNAseq marker | PREDICTED: protein trichome berefringence-like 7 |
| **colpYS_12469** | Illumina RNAseq marker | Complex 1 LYR protein |
| **colpYS_14201** | Illumina RNAseq marker | PREDICTED: probable arabinosyltransferase ARAD1 |
| **colpYS_15390** | Illumina RNAseq marker | uncharacterized protein |
| **colpYS_17495** | Illumina RNAseq marker | no homology |
| **colpYS_18199** | Illumina RNAseq marker | PREDICTED: protein trichome berefringence-like 7 |
| **colpYS_18468** | Illumina RNAseq marker | PREDICTED: phosphatidylinositol 3- and 4-kinase family protein |
| **colpYS_18982** | Illumina RNAseq marker | PREDICTED: filament-like plant protein |
| **colpYT_19195** | Illumina RNAseq marker | no homology |
| **colpYS_24813** | Illumina RNAseq marker | transcription factor bHLH |
| **colpYS_25642** | Illumina RNAseq marker | CLV1-like LRR receptor kinase |
| **colpYS_4053** | Illumina RNAseq marker | PREDICTED: TLC domain-containing protein 2 |
| **colpYS_4711** | Illumina RNAseq marker | no homology |
| **colpYT_50424** | Illumina RNAseq marker | no homology |
| **colpYS_7668** | Illumina RNAseq marker | CLV1-like LRR receptor kinase |
| **colpYS_8617** | Illumina RNAseq marker | PREDICTED: protein ROOT PRIMORDIUM DEFECTIVE 1 |
| **colpYS_8940** | Illumina RNAseq marker | hypothetical protein |
| E265 | homoloque from *S. latifolia* (Qiu et al. 2010) | FCLY (farnesylcysteine lyase) or prenylcysteine oxidase |
| oti193 | 454 sequencing marker from S. otites | RNA-binding S4 domain-containing protein |
| colpXS_20384 | Illumina RNAseq marker | no homology |
| colpXS_20972 | Illumina RNAseq marker | PREDICTED: NAC domain-containing protein 72-like |
| colpXS_10827 | Illumina RNAseq marker | PREDICTED: sulfate transporter 1.2 isoform X1 |
| colpXS_13981 | Illumina RNAseq marker | PREDICTED: transcription factor TGA3 |
| colpXS_14571 | Illumina RNAseq marker | hypothetical protein |
| colpXS_15384 | Illumina RNAseq marker | PREDICTED: probable mitochondrial adenine nucleotide transporter BTL3 isoform X1 |
| colpXS_17026 | Illumina RNAseq marker | PREDICTED: probable tRNA modification GTPase MnmE |
| colpXS_18134 | Illumina RNAseq marker | PREDICTED: Niemann-Pick C1 protein isoform X3 |
| colpXS_19384 | Illumina RNAseq marker | PREDICTED: 3-ketodihydrosphingosine reductase-like |
| colpXS_19736 | Illumina RNAseq marker | PREDICTED: branched-chain-amino-acid aminotransferase 2, chloroplastic isoform X2 |
| colpYS_13383 | Illumina RNAseq marker | PREDICTED: retinoblastoma-related protein isoform X1 |
| colpYS_14000 | Illumina RNAseq marker | PREDICTED: F-box protein At4g00755 |
| colpYS_14076 | Illumina RNAseq marker | no homology |
| colpYS_14647 | Illumina RNAseq marker | PREDICTED: probable alkaline/neutral invertase B |
| colpYS_15303 | Illumina RNAseq marker | PREDICTED: flap endonuclease GEN-like 1 |
| colpYS_16496 | Illumina RNAseq marker | PREDICTED: biogenesis of lysosome-related organelles complex 1 subunit 1 |
| colpYS_16597 | Illumina RNAseq marker | calcium-dependent protein kinase 16 |
| colpYS_17650 | Illumina RNAseq marker | PREDICTED: histone acetyltransferase of the MYST family 1 |
| colpYS_18193 | Illumina RNAseq marker | hypothetical protein |
| colpYS_19627 | Illumina RNAseq marker | PREDICTED: calcium-dependent protein kinase 11 |
| colpYS_19951 | Illumina RNAseq marker | PREDICTED: serine/threonine-protein phosphatase 7 long form homolog |
| colpYS_20903 | Illumina RNAseq marker | PREDICTED: serine/arginine-rich splicing factor RS31-like isoform X2 |
| colpYS_20980 | Illumina RNAseq marker | NAC2 |
| colpYS_21702 | Illumina RNAseq marker | xylulose kinase |
| colpYS_2485 | Illumina RNAseq marker | PREDICTED: hydroquinone glucosyltransferase |
| colpYS_25807 | Illumina RNAseq marker | PREDICTED: cullin-1 isoform X2 |

Note: The sequences showing complete sex linkage in the mapping population are in bold.
Labelling of the markers:
colpYT- markers found based on their male specific transcription (detected using LINKYX in Illumina RNAseq data). Two completely sex linked markers from this category show also male specific PCR amplification from genomic DNA.
colpYS - markers found based on the Y-linked SNP-polymorphism (detected using LINKYX in Illumina RNAseq data).
colpXS - markers found based on the X-linked SNP-polymorphism (detected using LINKYX in Illumina RNAseq data).
E265 – marker found based on the homology of the XY chromosome pair with the linkage group 6 in *S. latifolia* in numbering according to Papadopulos et al., 2015). This marker corresponds to the homologue E265 in *S. latifolia* (Qiu et al. 2010).

**Table S2: Analysis of the linkage disequilibrium at sexual phenotype and four tested loci.**

| population | sex linked locus | results of the the tests for the presence of the presumed W-linked alleles | | | |
|---|---|---|---|---|---|
| | | females | | males | |
| | | positive | negative | positive | negative |
| Kralovo Pole | otiW43556 | 12 | 0 | 0 | 18 |
| | otiW51286 | 12 | 0 | 0 | 18 |
| | otiW51403 | 12 | 0 | 0 | 18 |
| | otiW52979 | 12 | 0 | 0 | 18 |
| Rohatec | otiW43556 | 15 | 0 | 0 | 17 |
| | otiW51286 | 15 | 0 | 0 | 17 |
| | otiW51403 | 15 | 0 | 0 | 17 |
| | otiW52979 | 15 | 0 | 0 | 17 |

## Table S3: Overview of the analysis of the completely sex-linked sequences and their surroundings in *S. otites*.

As the order of the markers was unknown, the minimal size of the non-recombining region was calculated as the sum of the size of two smalest BACs plus the sum of the smallest distances from the contig ends in the two remaining BACs. According to this approach, the minimal size of the non-recombining region in S. otites is more than 180 kb.

| fully sex-linked gene | homology | number of BACs | Homologs of other genes in BACs | BAC size (kb) | smaller distance between the marker and the BAC end (kb) | larger distance between the marker and the BAC end (kb) |
|---|---|---|---|---|---|---|
| otiWT_51286 | leucine rich repeat receptor kinase protein | 4 | no other gene present | 120.4 | 25.2 | 27.5 |
| otiWT_52979 | pentatricopeptide (PPR) repeat-containing protein | 2 | wall associated kinase | more than 49.5* | 21.4 | 27.9 |
| otiWT_43556 | CRABS CLAW; zinc-finger motif containing protein; | 1 | no other gene present | 93 | 26.2 | 47.9 |
| otiWT_51403 | tetrapeptide repeat homeobox like protein | 3 | no other gene present | 99.1 | 12.6 | 38.7 |

* As the complete assembly was not possible in the BAC carrying otiW52979, its minimal length is given as the lenth of the largest contig.

**Table S4A: Analysis of combinations of the ancestral states of sex determining system in the most recent common ancestors of the section Otites and in the MRCA of the group Otites in BayesTraits (S. pseudotites set as Y).** First letter corresponds to ancestral state in the MRCA of the section Otites and the second to the ancestral state in the MCA of the group Otites (W-female heterogamety, N-non-dioecy, Y-male heterogamety).

Note: * = positive evidence: 2 < 2×difference in ln(marginal likelihood) > 6, ** = strong evidence: 6 < 2×difference in ln(marginal likelihood) > 10, *** = very strong evidence: 10 < 2×difference in ln(marginal likelihood) (Kass & Raftery 1995)

| Combinations of the ancestral states | Ln (marginal likelihood) | 2×differences in ln(marginal likelihood) | | | | | | | | |
|---|---|---|---|---|---|---|---|---|---|---|
| | | -WW | -YY | -WY | -NW | -NY | -YW | -NN | -WN | -YN |
| WW | -10.388936 | 0.000 | 3.255* | 5.279* | 5.626* | 6.763** | 9.025** | 9.269** | 14.867*** | 19.810*** |
| YY | -12.016426 | -3.255 | 0.000 | 2.024* | 2.371* | 3.508* | 5.770* | 6.014** | 11.612*** | 16.555*** |
| WY | -13.028208 | -5.279 | -2.024 | 0.000 | 0.347 | 1.484 | 3.746* | 3.990* | 9.589** | 14.532*** |
| NW | -13.201756 | -5.626 | -2.371 | -0.347 | 0.000 | 1.137 | 3.399* | 3.642* | 9.242** | 14.185*** |
| NY | -13.770253 | -6.763 | -3.508 | -1.484 | -1.137 | 0.000 | 2.262* | 2.506* | 8.105** | 13.048*** |
| YW | -14.901343 | -9.025 | -5.770 | -3.746 | -3.399 | -2.262 | 0.000 | 0.244 | 5.842* | 10.786*** |
| NN | -15.023221 | -9.269 | -6.014 | -3.990 | -3.643 | -2.506 | -0.244 | 0.000 | 5.599* | 10.542*** |
| WN | -17.822585 | -14.867 | -11.612 | -9.589 | -9.242 | -8.105 | -5.842 | -5.599 | 0.000 | 4.943* |
| YN | -20.294095 | -19.810 | -16.555 | -14.532 | -14.185 | -13.048 | -10.786 | -10.542 | -4.943 | 0.000 |

**Table 4B: Analysis of combinations of the ancestral states of sex determining system in the most recent common ancestors of the section Otites and in the MRCA of the group Otites in BayesTraits (S. pseudotites set as WY).** First letter corresponds to ancestral state in the MRCA of the section Otites and the second to the ancestral state in the MCA of the group Otites (W-female heterogamety, N-non-dioecy, Y-male heterogamety).

Note: * = positive evidence: 2 < 2×difference in ln(marginal likelihood) > 6, ** = strong evidence: 6 < 2*difference in ln(marginal likelihood) > 10, *** = very strong evidence: 10 < 2*difference in ln(marginal likelihood) (Kass & Raftery 1995)

| Combinations of the ancestral states | Ln (marginal likelihood) | 2×differences in ln(marginal likelihood) | | | | | | | | |
|---|---|---|---|---|---|---|---|---|---|---|
| | | -WW | -NW | -YY | -YW | -WY | -NN | -NY | -WN | -YN |
| WW | -7.602 | 0.000 | 5.665* | 8.220** | 9.260** | 10.341*** | 11.733*** | 11.787*** | 17.337*** | 22.811*** |
| NW | -10.434 | -5.665 | 0.000 | 2.555* | 3.595* | 4.676* | 6.068** | 6.122** | 11.672*** | 17.145*** |
| YY | -11.712 | -8.220 | -2.555 | 0.000 | 1.040 | 2.121* | 3.513* | 3.567* | 9.117** | 14.590*** |
| YW | -12.232 | -9.260 | -3.595 | -1.040 | 0.000 | 1.081 | 2.473* | 2.527* | 8.077** | 13.550*** |
| WY | -12.772 | -10.341 | -4.676 | -2.121 | -1.081 | 0.000 | 1.392 | 1.446 | 6.996** | 12.469*** |
| NN | -13.468 | -11.733 | -6.068 | -3.513 | -2.473 | -1.392 | 0.000 | 0.054 | 5.604* | 11.077*** |
| NY | -13.495 | -11.788 | -6.122 | -3.567 | -2.527 | -1.446 | -0.054 | 0.000 | 5.550* | 11.023*** |
| WN | -16.270 | -17.337 | -11.672 | -9.117 | -8.077 | -6.996 | -5.604 | -5.550 | 0.000 | 5.473* |
| YN | -19.007 | -22.811 | -17.145 | -14.590 | -13.550 | -12.469 | -11.077 | -11.023 | -5.473 | 0.000 |

## Table S5: Overview of the plant species and samples used in analyses.

| Species | voucher or collector | Geographic origin | RNAseq origin |
|---|---|---|---|
| *Silene baschkirorum Janisch.* | BRNU 590993 | Muradym (previous name Yuldybaevo 3-e village; Mrakovo distr., Bashkortostan Republic, Russian Federation) | this manuscript |
| *Silene borysthenica (Gruner) Walters* | BRNU 576274 | eastern periphery of Abakan City (Khakasia, Minusinsk Basin; Russian Federation) | this manuscript |
| *Silene colpophylla Wrigley* | Janousek IBPBR 27 | originally cultivated at ENS Lyon, Lyon, France | this manuscript |
| *Silene nocturna L.* | BRNU615725 | Kalyviani (distr. Chaniá Gramvousa Peninsula, Crete) | this manuscript |
| *Silene nutans L.* | Bengt Oxelman, University of Gothenburgh, Sweden | | this manuscript |
| *Silene otites Sm.* | Janousek IBPBR 1 | Nantes (Loire-Atlantique region, France) | this manuscript |
| *Silene paradoxa L.* | Deborah Triant, University of Virginia, USA | cultivated at university of Virginia, USA | publically available, BioSample: SAMN02351094; |
| *Silene pseudotites Besser ex Reichenb.* | Janousek IBPBR 55 | Basovizza (municipality of Trieste, Province of Trieste, Italy) | this manuscript |
| *Silene sibirica (L.) Pers.* | BRNU631315 | Krasnogorka village (Omsk region, Poltavka district, Russian Federation) | this manuscript |
| *Silene wolgensis Otth* | BRNU 590996 | Novoaleksandrovka village (18 km SW of Zilair) (Bashkortostan Republic, Zilairskoe plateau, Zilair distr.; Russian Federation) | this manuscript |

**Table S6: List of the accession numbers of the NCBI BioProjects storing the RNAseq data.**

| Species | TaxId | BioProject ID | type of data |
|---|---|---|---|
| *Silene baschkirorum* | 1390714 | PRJNA407407 | Transcriptome Illumina |
| *Silene borysthenica* | 1390715 | PRJNA404055 | Transcriptome Illumina |
| *Silene colpophylla* | 512022 | PRJNA361444 | Transcriptome Illumina |
| *Silene otites* | 42039 | PRJNA403783 | Transcriptome 454 |
| *Silene otites* | 42039 | PRJNA401641 | Transcriptome Illumina |
| *Silene pseudotites* | 42041 | PRJNA407280 | Transcriptome Illumina |
| *Silene sibirica* | 1291645 | PRJNA407314 | Transcriptome Illumina |
| *Silene wolgensis* | 1389442 | PRJNA407297 | Transcriptome Illumina |

# Table S7: Overview of the Illumina RNAseq sequenced samples studied by LINKYX

**Silene otites**

| type of specimen | sample_ ID | lane_ID | raw reads (left+right ) | trimmed reads (paired, left + right) | reference: father flower buds | | | reference: mother + daugthers (flower buds) | | |
|---|---|---|---|---|---|---|---|---|---|---|
| | | | | | mapped | unmapped | % mapped | mapped | unmapped | % mapped |
| mother-leaves | NNN18L | OSR13s005080 | 70954976 | 60475858 | 55884502 | 4591356 | 92% | 57454103 | 3021755 | 95% |
| mother-flower buds | NNN18P | OSR13s005080 | 63497510 | 53753330 | 49164565 | 4588765 | 91% | 50595473 | 3157857 | 94% |
| father-leaves | NNN27L | OSR13s005080 | 63351950 | 53240266 | 49471205 | 3769061 | 93% | 50193590 | 3046676 | 94% |
| father-flower buds | NNN27P | OSR13s005080 | 73789112 | 62611828 | 58666877 | 3944951 | 94% | 57799122 | 4812706 | 92% |
| daughters-flower buds | NNN18x27_11FP | OSR14s007369 | 50281150 | 42796220 | 40311011 | 2485209 | 94% | 40628257 | 2167963 | 95% |
| | NNN18x27_12FP | OSR14s007369 | 76914222 | 67953504 | 62359183 | 5594321 | 92% | 64241110 | 3712394 | 95% |
| | NNN18x27_15FP | OSR14s007369 | 82893462 | 73432536 | 68794151 | 4638385 | 94% | 69625097 | 3807439 | 95% |
| | NNN18x27_1FP | OSR14s007369 | 50704846 | 46292274 | 43570561 | 2721713 | 94% | 44088454 | 2203820 | 95% |
| | NNN18x27_2FP | OSR15s008719 | 114110562 | 108218530 | 100418719 | 7799811 | 93% | 101745376 | 6473154 | 94% |
| | NNN18x27_31FP | OSR15s008719 | 116844498 | 109860928 | 100575169 | 9285759 | 92% | 102626178 | 7234750 | 93% |
| sons-flower buds | NNN18x27_14MP | OSR15s008719 | 121084668 | 114160060 | 102000059 | 12160001 | 89% | 104747391 | 9412669 | 92% |
| | NNN18x27_17MP | OSR14s007369 | 99980922 | 77105218 | 70444017 | 6661201 | 91% | 71303848 | 5801370 | 92% |
| | NNN18x27_19MP | OSR14s007369 | 59486732 | 52122456 | 48561166 | 3561290 | 93% | 48395139 | 3727317 | 93% |
| | NNN18x27_1BMP | OSR15s008719 | 84209270 | 79267464 | 72909619 | 6357845 | 92% | 73570393 | 5697071 | 93% |
| | NNN18x27_25MP | OSR15s008719 | 74850408 | 70653560 | 65092312 | 5561248 | 92% | 65243526 | 5410034 | 92% |
| | NNN18x27_32MP | OSR15s008719 | 88160726 | 83586110 | 76393882 | 7192228 | 91% | 76792548 | 6793562 | 92% |

**Silene borysthenica**

| type of specimen | sample_ ID | lane_ID | raw reads (left+right ) | trimmed reads (paired, left + right) | reference: father flower buds | | | reference: mother + daugthers (flower buds) | | |
|---|---|---|---|---|---|---|---|---|---|---|
| | | | | | mapped | unmapped | % mapped | mapped | unmapped | % mapped |
| mother-flower buds | BOR_5FP | OSR13s005080 | 70986938 | 60535752 | 51405283 | 9130469 | 85% | 57394158 | 3141594 | 95% |
| father-leaves | NEW_BOR_3L | OSR14s007368 | 46870138 | 34770910 | 31521559 | 3249351 | 91% | 32654613 | 2116297 | 94% |
| father-flower buds | NEW_BOR_3P | OSR14s007368 | 75799902 | 64518596 | 60130677 | 4387919 | 93% | 56852092 | 7666504 | 88% |
| daughters-flower buds | BB_57FP | OSR16s010836 | 142012050 | 112572718 | 100018589 | 12554129 | 89% | 107266440 | 5306278 | 95% |
| | BB_117FP | OSR16s010836 | 129036354 | 101173034 | 91547167 | 9625867 | 90% | 96121690 | 5051344 | 95% |
| | BB_122FP | OSR16s010836 | 126438494 | 100712834 | 92183499 | 8529335 | 92% | 96217456 | 4495378 | 96% |
| | BB_126FP | OSR16s010836 | 112921298 | 87638264 | 79320530 | 8317734 | 91% | 83359904 | 4278360 | 95% |
| | BB_13XFP | OSR16s010836 | 132560834 | 104558974 | 95962601 | 8596373 | 92% | 99992934 | 4566040 | 96% |
| sons-flower buds | BB_1MP | OSR15s008724 | 91021474 | 85778596 | 74860355 | 10918241 | 87% | 77863602 | 7914994 | 91% |
| | BB_4MP | OSR15s008724 | 122179108 | 114319088 | 99774242 | 14544846 | 87% | 103992581 | 10326507 | 91% |
| | BB_6MP | OSR15s008724 | 101449614 | 93813624 | 84592968 | 9220656 | 90% | 83182051 | 10631573 | 89% |
| | BB_7MP | OSR15s008724 | 87475176 | 82419008 | 72895390 | 9523618 | 88% | 74286784 | 8132224 | 90% |
| | BB_12MP | OSR15s008724 | 62164452 | 58309236 | 51627754 | 6681482 | 89% | 51984175 | 6325061 | 89% |
| | BB_108MP | OSR16s010836 | 103852006 | 80710546 | 73588918 | 7121628 | 91% | 73147941 | 7562605 | 91% |
| | BB_133MP | OSR16s010836 | 125729588 | 94446450 | 84236253 | 10210197 | 89% | 86462796 | 7983654 | 92% |

**Silene colpophylla**

| type of specimen | sample_ ID | lane_ID | raw reads (left+right ) | trimmed reads (paired, left + right) | reference: mother flower buds | | | reference: father (leaves) + sons (flower buds) | | |
|---|---|---|---|---|---|---|---|---|---|---|
| | | | | | mapped | unmapped | % mapped | mapped | unmapped | % mapped |
| mother-leaves | AL | OSR14s007366 | 81365452 | 71020128 | 68219559 | 2800569 | 96% | 65094996 | 5925132 | 92% |
| mother-flower buds | AP | OSR14s007366 | 70002736 | 58695892 | 55589384 | 3106508 | 95% | 54711208 | 3984684 | 93% |
| father-leaves | BL | OSR14s007366 | 59077754 | 49428900 | 21258152 | 28170748 | 43% | 22997607 | 26431293 | 47% |
| uncle-leaves | A1_L | OSR13s005080 | 62309456 | 62606496 | 56590057 | 6016439 | 90% | 58832677 | 3773819 | 94% |
| uncle-flower buds | A1_P | OSR13s005080 | 82168286 | 69900880 | 59609438 | 10291442 | 85% | 65008062 | 4892818 | 93% |
| daughters-flower buds | AxB_8P | OSR14s007367 | 53680064 | 47620884 | 44313713 | 3307171 | 93% | 44359826 | 3261058 | 93% |
| | AxB_9P | OSR14s007368 | 59559668 | 49369106 | 46082392 | 3286714 | 93% | 46182804 | 3186302 | 94% |
| | AxB_15P | OSR14s007368 | 73420962 | 59894500 | 55775767 | 4118733 | 93% | 55802174 | 4092326 | 93% |
| | AxB_18P | OSR14s007368 | 36737780 | 29862268 | 27673614 | 2188654 | 93% | 27910452 | 1951816 | 93% |
| | AxB_26P | OSR14s007368 | 37950966 | 30356706 | 28011562 | 2345144 | 92% | 28241805 | 2114901 | 93% |
| | AxB_53P | OSR14s007368 | 83923538 | 69234046 | 64033971 | 5200075 | 92% | 64311453 | 4922593 | 93% |
| sons-flower buds | AxB_6P | OSR14s007367 | 67003158 | 59874552 | 51216116 | 8658436 | 86% | 56401754 | 3472798 | 94% |
| | AxB_10P | OSR14s007367 | 48473938 | 43502350 | 38736084 | 4766266 | 89% | 40496151 | 3006199 | 93% |
| | AxB_11P | OSR14s007367 | 45500730 | 38189340 | 32497723 | 5691617 | 85% | 35701260 | 2488080 | 93% |
| | AxB_12P | OSR14s007367 | 64401312 | 58337356 | 47266653 | 11070703 | 81% | 54572511 | 3764845 | 94% |
| | AxB_45P | OSR14s007367 | 69519626 | 62606496 | 55847056 | 6759440 | 89% | 58554164 | 4052332 | 94% |
| | AxB_46P | OSR14s007367 | 55960836 | 50045002 | 44356247 | 5688755 | 89% | 46888865 | 3156137 | 94% |
